# Perspectives on the Catalytic Processes for the Deep Valorization of Carbohydrates into Fuels and Chemicals

**DOI:** 10.3390/molecules30173498

**Published:** 2025-08-26

**Authors:** Aigul T. Zamanbekova, Alima K. Zharmagambetova, Assemgul S. Auyezkhanova, Eldar T. Talgatov, Aigul I. Jumekeyeva, Sandugash N. Akhmetova, Alima M. Kenzheyeva

**Affiliations:** Laboratory of Organic Catalysis, D.V. Sokolsky Institute of Fuel, Catalysis, and Electrochemistry, Kunaev Str. 142, Almaty 050010, Kazakhstan; a.zharmagambetova@ifce.kz (A.K.Z.); a.auezkhanova@ifce.kz (A.S.A.); e.talgatov@ifce.kz (E.T.T.); a.dzhumekeeva@ifce.kz (A.I.J.); s.akhmetova@ifce.kz (S.N.A.); a.kenzheeva@ifce.kz (A.M.K.)

**Keywords:** ruthenium catalysts, biomass, one-pot conversion, platform chemicals, hydrogenation

## Abstract

The global depletion of fossil resources, combined with accelerating climate change and environmental concerns, is driving intensive research into alternative, sustainable sources of energy and raw materials. Particular attention is being paid to lignocellulosic biomass as the most abundant and renewable organic resource. The catalytic conversion of biomass-derived carbohydrates into high-value-added products (fuels and chemicals) aligns with the principles of sustainable development and offers a viable alternative to petroleum-based feedstocks. This review provides a product-oriented perspective on the deep valorization of carbohydrates, focusing on catalytic strategies that enable the production of renewable fuels and chemicals. It highlights two key stages in the valorization of lignocellulosic biomass: (1) the acid-catalyzed conversion of carbohydrates into platform molecules (furfural, 5-hydroxymethylfurfural, and levulinic acid); and (2) the selective hydrogenation and hydrogenolysis of these intermediates to obtain target end products. These target products fall into two major categories: (i) biofuels and fuel additives; and (ii) green chemicals, such as solvents, pharmaceuticals, agrochemicals, cosmetics, and intermediates for the synthesis of biobased polymeric materials, including polyesters, resins, and polyurethanes. Particular emphasis is placed on recent advances in the development of heterogeneous catalysts. Solid acid catalysts used in the synthesis of platform molecules are discussed, along with ruthenium-based catalysts employed in the subsequent hydrogenation and hydrogenolysis steps. Recent efforts toward integrating both catalytic stages into a single one-pot processes using bifunctional metal–acid catalysts and dual catalytic systems based on ruthenium are also reviewed, as they represent a promising route to simplify biomass valorization schemes and improve product selectivity toward fuels and chemicals.

## 1. Introduction

The continuous depletion of conventional fossil fuel resources and the risk of global climate change have directed global attention toward biomass as a potential sustainable source of energy and organic chemicals that can gradually replace petroleum [1]. Lignocellulosic biomass mainly composed of cellulose (35–50 wt%), hemicellulose (20–30 wt%), and lignin (10–25 wt%) can be converted into fuels and valuable chemicals using various routes such as thermochemical (gasification, pyrolysis, and liquefaction), biochemical (fermentation by anaerobic digestion), and chemocatalytic (hydrolysis, dehydration, and hydrogenation) methods [2,3].

Among these routes, chemocatalytic valorization through a two-step sequence of (i) the acid-catalyzed conversion of carbohydrates into platform molecules followed by (ii) metal-catalyzed hydrogenation/hydrogenolysis is particularly promising. This approach combines the advantages of both thermochemical and enzymatic strategies, offering fast reaction rates, high selectivity, and compatibility with a wide range of carbohydrate feedstocks under relatively mild and energy-efficient conditions [3,4]. Moreover, chemocatalytic methods enable the selective transformation of the multiple functional groups in carbohydrate molecules, providing access to a diverse range of target compounds with tailored properties [4].

In the first step, in the presence of acidic catalysts, cellulose and hemicellulose can be depolymerized into C6 (glucose and fructose) and C5 (arabinose, galactose, and xylose) monosaccharide sugars, respectively [3]. These sugars can undergo further acid-catalyzed dehydration to form key platform molecules such as 5-hydroxymethylfurfural (5-HMF), levulinic acid (LA), and furfural (FUR) etc. [4,5,6]. These intermediates are highly unsaturated compounds, containing C=C and C=O bonds as well as a furan ring, and can be efficiently transformed in the second catalytic step via metal-catalyzed hydrogenation or hydrogenolysis. This two-step chemocatalytic strategy enables the production of a wide range of renewable products, which can be classified into two principal categories: (i) biofuels and fuel additives, and (ii) green chemicals, including solvents, agrochemicals, pharmaceuticals, printing inks, cosmetics, and monomers for the synthesis of biobased polymeric materials such as polyesters, polyurethanes, and resins [5,7,8,9].

Noble metal (Pd, Pt, and Ru)-supported catalysts exhibit high hydrogenation activity in the liquid phase due to their ability to adsorb both hydrogen and unsaturated substrates [9]. Among them, Ru-based catalysts are considered as the most promising for the hydrogenation of a wide variety of biomass-derived compounds due to their superior activity compared to other noble metal catalysts [3]. Recent scarce comprehensive reviews on the Ru-catalyzed hydrogenation of furfural and levulinic acid showed that various solid supports such as carbon-based materials, inorganic oxides, and metal–organic frameworks are used in design of the supported catalysts with improved catalytic performance [10,11,12]. These studies emphasize that the nature of the support significantly affects the catalysts properties. Mesoporous materials with high surface area and containing electron donor centers can provide the suitable dispersion, size, and electronic properties of Ru particles. Moreover, acid sites on the surface of supports can play a crucial role in the performance and selectivity of the catalysts [12,13,14].

Recently, bifunctional metal–acid catalysts have attracted significant attention. In these systems, the hydrogenation function provided by the metal component (e.g., Ru, Pt, Pd) is combined with the acidic properties of the support (e.g., zeolites, γ-Al_2_O_3_, SiO_2_, ZrO_2_, etc.) [13,14]. This combination allows the simultaneous catalysis of hydrogenation reactions and acid-catalyzed transformations such as dehydration in a single step. Such synergy opens new opportunities for one-pot transformations of carbohydrates into high-value-added downstream products. However, only a limited number of studies have been devoted to this issue. Despite recent advances, further research is needed to design more efficient and selective bifunctional catalysts that enable integrated, one-pot processes with minimal waste and energy input. The ultimate goal in this field is to establish sustainable and economically viable catalytic platforms capable of replacing petroleum-derived processes with biomass-based alternatives at an industrial scale.

This review focuses on the deep valorization of biomass into high-value-added products, particularly renewable fuels and specialty chemicals, via a two-stage catalytic sequence. The first stage involves the acid-catalyzed transformation of carbohydrates into platform molecules (5-HMF, LA, and FUR), while the second involves their Ru-catalyzed hydrogenation and hydrogenolysis. Recent advances in the development of bifunctional catalysts, enabling the integration these steps into a single catalytic process, are also partly discussed.

## 2. Conversion of Carbohydrates into Platform Molecules

### 2.1. Furfural Production

Furfural (FUR) is an important platform chemical compound that is widely used in the synthesis of a range of valuable chemical compounds (biofuels, solvents, polymers, and fine chemical products) [15,16,17,18].

**Figure 1:**
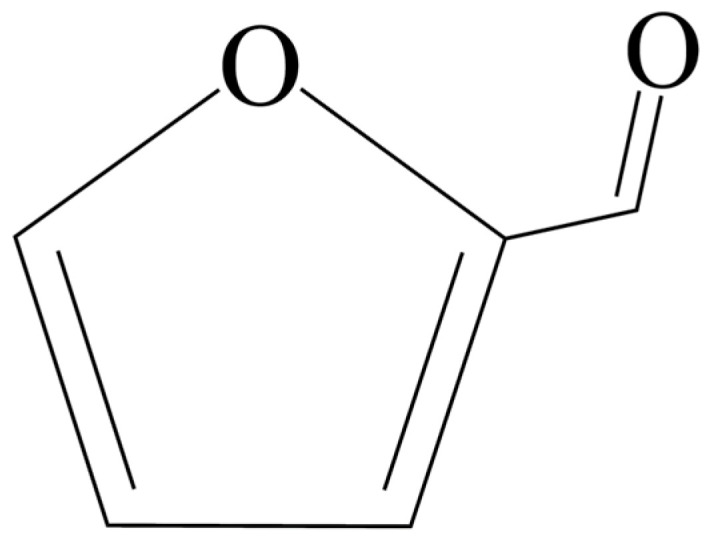
Structural formula of furfural.

FUR (C_5_H_4_O_2_) is an aromatic aldehyde with a furan ring and an aldehyde group (Figure 1). Due to the presence of a furan ring and a reactive aldehyde group, furfural easily reacts in reduction, aldol condensation, and hydrogenation reactions.

**Figure 2:**
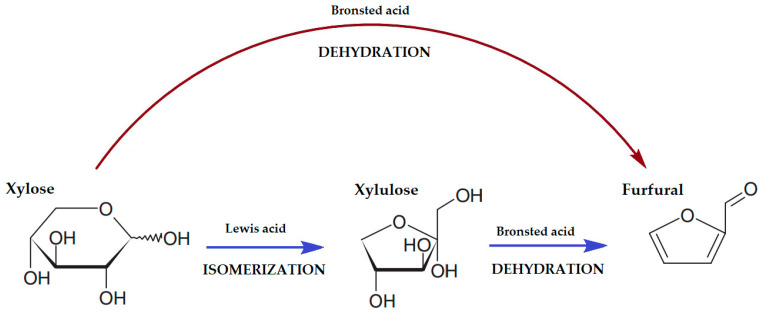
Pathways of furfural production: direct (brown) and indirect (blue).

The commercial production of furfural was first developed by Quaker Oats Technology in the 1920s in the United States [19]. FUR is derived mainly from pentosanes contained in the hemicellulose of lignocellulosic biomass. Generally, furfural is produced at a temperature range of 150 to 200 °C in organic solvents using H_2_SO_4_ as a catalyst [20]. The production mechanism of furfural has been described in detail in many studies in recent years [21,22]. The two most common pathways for the conversion of pentose (xylose) to FUR are known: direct dehydration in the presence of Bronsted acid and the isomerization of xylose to xylulose using Lewis acid followed by dehydration to FUR using Bronsted acid (Figure 2) [22,23]. The second route gives fewer side reactions and proceeds under milder conditions compared to direct dehydration and provides higher yields of furfural [22,24].

Recent studies show that high process efficiency is achieved with the correct selection of a catalyst capable of providing both substrate activation and selective dehydration of C5-sugars. Homogeneous and heterogeneous catalysts are used as catalysts for the production of furfural. Homogeneous catalysts include mineral acids [16,25,26], metal salts [26,27,28], heterogeneous catalysts such as zeolites [29,30], solid phosphates [31,32], carbon based catalysts [33,34,35], etc. However, homogeneous catalysts have a range of disadvantages related to utilization, equipment corrosion, and environmental pollution. Heterogeneous acid systems with good stability, regeneration, and recyclability are considered as an alternative to homogeneous catalysts.

#### 2.1.1. Carbon-Based Catalysts

Carbon-based solid catalysts have advantages such as rich pore structure, high hydrothermal stability, stability under repeated cycling, easy recovery, low cost, and low environmental impact [33,34,35,36]. Due to these advantages, carbon-based solid catalysts have been widely used in the catalytic conversion of monosaccharides to furfural (FUR) [35,37]. Antonyraj et al. [38] developed a sulfocarbon-based catalyst for the dehydration of xylose to furfural in a two-phase solvent system of water–methyl isobutyl ketone. The catalyst, obtained by the acidification of carbon derived from crude lignin (LC) with 1 N H_2_SO_4_, demonstrated 100% xylose conversion present in the acid pretreatment liquid, with a furfural yield of 60% under reaction conditions of 175 °C and 20 bar pressure for 3 h. Yang et al. [34] reported a novel solid carbon-based acidic catalyst (SC-GCa-800), prepared by carbonizing calcium gluconate at high temperature, followed by sulfonation with 4-diazoniumbenzenesulfonate at room temperature. SC-GCa-800 was demonstrated to be an efficient solid acid catalyst for furfural production from xylose in 1,4-dioxane. A furfural yield of 76.9% was achieved from 100 mg of xylose at 140 °C in 40 min using 50 mg of the catalyst. The catalyst exhibited high stability and could be reused up to five times without significant loss of catalytic activity.

Zhou et al. [35] developed a solid acid carbon-based catalyst for the two-step conversion of lignocellulosic biomass to furfural. The catalyst was prepared by mixing glucose with various amounts of Zn(NO_3_)_2_·6H_2_O, followed by drying and carbothermal reduction at 750 °C under a nitrogen atmosphere. The resulting carbon material (ZnC-x) was then sulfonated with concentrated sulfuric acid at 150 °C for 6 h. The catalysts were labeled ZnCS-1, ZnCS-2, and ZnCS-3 according to their zinc content. ZnCS-2 showed the best performance, achieving a furfural yield of 72.4% at 180 °C in 60 min with 10 wt% catalyst loading.

In [39], a magnetic carbon-based solid acid (MCSA) catalyst was synthesized via the pyrolysis of tobacco stalks impregnated with FeCl_3_, followed by the acidification of the resulting magnetic carbon material with sulfuric acid. The MCSA catalyst contained 5.04 μmol/mg of Bronsted acid sites (carboxyl groups) and 21.16 μmol/mg of Lewis acid sites, both contributing to its high catalytic efficiency in converting tobacco stalk biomass to furfural in a water–γ-valerolactone solvent system. A maximum furfural yield of 46.68% was achieved at 190 °C after 120 min. Liu et al. [40] synthesized a series of carbon-supported metal oxide catalysts (M@FRC, M = Sn, Al, Fe, Zn, and Mg) by the simple incipient-wetness impregnation approach. In this approach, furfural residue (FUR), composed of cellulose (29.56 ± 0.5 wt%) and lignin (29.56 ± 0.5 wt%), was used as a natural carbon building block. The catalysts were tested in the dehydration of xylose to furfural in the aqueous medium. The results showed that the prepared carbon-supported metal oxide catalysts present efficient catalytic performance for the dehydration of xylose to furfural in the aqueous media, and a high xylose conversion (82.3–96.7%) and FUR yield (28.7–38.3%) were obtained at 180 °C for 1.5 h in aqueous medium.

Yusuff et al. [41] employed sulfonated carbon catalysts, prepared from eucalyptus-derived activated carbon (EAC) and sulfonating agents such as H_2_SO_4_ and p-toluenesulfonic acid (TsOH), for the dehydration of xylose to furfural using γ-valerolactone as a green solvent. A maximum furfural yield of 74.61% was achieved at 180 °C with a γ-valerolactone-to-xylose ratio of 3 mL/g. Qiu et al. [37] prepared a biochar-based catalyst functionalized with phosphotungstic acid via a one-step hydrothermal method and employed it for the conversion of xylose to FUR. Physicochemical characterization revealed that the catalyst possessed a high density of functional groups and featured both Bronsted and Lewis acid sites. The highest furfural yield (81.02%) was achieved at 180 °C with a reaction time of 20 min in the presence of the synthesized catalyst.

Xu et al. [33] developed a new carbon-based solid acid catalyst (S-800-CG) using the high-temperature carbonization of calcium gluconate followed by sulfonation with 4-diazoniumbenzenesulfonate at room temperature to convert xylose or corn cob to furfural by a one-step process. The effects of carbon support, calcination temperature, reaction temperature, reaction temperature, residence time, catalyst loading, and substrate concentration were studied; S-800-CG demonstrated high catalytic efficiency for furfural production from xylose (50 mg) in 1,4-dioxane, achieving an 85.9% yield at 140 °C in 40 min using 100 mg of the catalyst. Additionally, a furfural yield of 52.9% was obtained from 200 mg of corncob at 190 °C in 70 min under the same catalyst loading.

Ma et al. [42] developed a sulfonated graphene-based catalyst that exhibited high catalytic activity for the conversion of xylose and corn stalk into furfural. A furfural yield of 96% was achieved from xylose at 150 °C in 40 min. In the case of corn stalk, a furfural yield of 48% was obtained at 190 °C in 50 min.

In general, a comparative analysis of the research on the development of carbon-based catalysts showed that carbon is a promising material for the development of efficient catalysts for use in the production of furfural.

#### 2.1.2. Zeolite-Based Catalysts

One of the brightest representatives of heterogeneous acid catalysts is zeolite. The use of porous solid acid catalysts instead of mineral acids is attractive [43,44,45,46]. Zeolite catalysts are of great interest in terms of production cost and availability [43]. Bruce et al. [43] investigated zeolites with small pores as catalysts for the dehydration of xylose and biomass to furfural (FUR) in a monophasic γ-valerolactone/water system. Although the pore sizes were much smaller than the kinetic diameter of the sugars, the yield of furfural from xylose on the commercial SAPO-34 catalyst was 40%. The SAPO-34 catalyst was recycled many times with only a 5% reduction in furfural yield. No significant leaching of acid sites was observed.

Gallo et al. [44] reported the preparation of furfural from C5 monosaccharides (xylose, arabinose, and ribose) and from real biomass (corn fiber) using zeolite H-Beta as a catalyst in a monophase system containing γ-valerolactone. A higher yield of furfural was obtained from arabinose and was 73% using H-Beta. In the presence of sulfuric acid and mordenite, the yield of furfural was 44 and 49%, respectively. The yield of furfural from corn fiber using H-Beta, H-Mordenite, and sulfuric acid was 62, 44, and 55%, respectively.

Gao et al. [30] obtained furfural from a spent aqueous solution of hemicellulose derived from hardwood kraft pulp by an environmentally friendly method. A maximum furfural yield of 82.4% and xylose conversion of 96.8% were achieved at 463 K, in the presence of 1.0 g ZSM-5, 1.05 g NaCl, and an organic solvent-to-aqueous-phase ratio of 30:15 for 3 h. In contrast, using a pure xylose solution of the same concentration resulted in a significantly lower furfural yield of 51.5%. Moreover, the catalyst demonstrated good stability, with only a slight decrease in furfural yield to 67.1% after five reuse cycles.

The use of traditional zeolite catalysts for the conversion of xylose to furfural has a number of limitations such as low yield of the target product, the possibility of tar formation, etc. One of the solutions to these disadvantages is zeolite modification. Appropriate acid treatment of zeolites can provide them with a large number of pores, which can facilitate the easy separation of product molecules from the inner surface of aluminosilicate, thereby preventing tar formation and carbon accumulation, resulting in higher activity and selectivity [45].

Wang et al. [45] synthesized a Cr-deAl-Y solid acid catalyst by the modification of zeolite H-Y. The modified catalyst was used to catalyze the conversion of xylose to furfural in the water/n-butanol system. The maximum furfural yield reached 77.5%, with a xylose conversion of 99.7% at 180 °C in 30 min

Lima et al. [29] studied the cyclodehydration of *d*-xylose to FUR in the aqueous phase at 170 °C in the presence of a composite material based on Beta zeolite nanocrystals embedded in silicon mesoporous matrix TUD-1 (BEATUD). After 8 h, the xylose conversion reached 98% and the FUR yield was 74%. It is known that the disadvantage of using zeolite catalysts is the formation of coke, which can lead to a decrease in FUR selectivity, especially at high conversions. According to the authors of this study, reducing the size of catalyst crystallites to nanosize led to an increase in the ratio of surface area to micropore volume and a reduction in the length of diffusion paths, which allowed to avoid strong coke formation. In addition, reducing the crystallite size can enhance the ratio of Lewis and Bronsted acid sites, which may lead to an increase in FUR selectivity [29].

In [46], HZ-Na free-m and HZ-Na-m zeolites were prepared without or with the addition of Na^+^ ions to the mother gels. The conversion of xylose to furfural was carried out at 150 °C for 60 min. The maximum furfural yield (63.0%) and selectivity (64%) were obtained on HZ-Na-50 catalyst. Hoang et al. [47] investigated the catalytic activity of sulfonated zeolite ZSM-5 and a highly tunable co-solvent system in the co-production of 5-HMF and furfural from lignocellulosic biomass in a single-phase reaction process by combining biomass hydrolysis with the catalytic dehydration of sugars. The sulfonated zeolite ZSM-5 showed high catalytic activity for the conversion of corn cob biomass to 5-HMF and furfural. The reaction conditions including solvent, catalyst loading, temperature, and reaction time were investigated. In optimal conditions, the furfural yield was 89%.

Thus, the different modification of zeolites promotes an increase in the activity and selectivity of catalytic systems, preventing the formation of resins and carbon accumulation.

#### 2.1.3. Other Solid Acid Catalysts

Solid acid materials based on metal oxides (ZrO_2_, SnO_4_, TiO_2_, and Nb_2_O_5_) or mesoporous silica, containing Bronsted and/or Lewis acid sites, can also serve as efficient catalysts for the dehydration of carbohydrates into furfural [48,49,50].

In [51], a novel solid acid catalyst (SnO_x_-NPs@SC) with tunable Lewis/Bronsted acidity was developed by combining tin oxide nanoparticles with sulfonated carbon microspheres for the conversion of glucose and xylose to 5-hydroxymethylfurfural (5-HMF) and furfural (FUR), respectively. The optimized catalyst (0.35-SnO_x_-NPs@SC-500) exhibited excellent catalytic performance, achieving a 5-HMF yield of 83.2% at 180 °C in 2 h and a FUR yield of 80.8% at 170 °C in 2 h. Furthermore, the catalyst exhibited good stability, maintaining its performance over five consecutive reaction cycles.

Fúnez-Núñez et al. [52] used γ-Al_2_O_3_ as a solid acid catalyst for the dehydration of xylose in the two-phase water/toluene system. The addition of alkaline earth metal chlorides promoted the ease of furfural extraction from the aqueous phase. In the presence of CaCl_2_ and γ-Al_2_O_3_, the furfural yield was 83% after 50 min at 175 °C. The γ-Al_2_O_3_ did not lose activity during 10 catalytic cycles without any pretreatment. In [53], solid acid catalysts based on niobium oxide supported on various materials—including commercial fumed silica, γ-Al_2_O_3_, MCM-41, and SBA-15—were synthesized and evaluated for the dehydration of D-xylose to furfural. Both monophasic (water) and biphasic (water/toluene) systems were tested, showing comparable xylose conversion but significantly enhanced furfural selectivity when toluene was used as a co-solvent. The γ-Al_2_O_3_-supported catalyst (Al-12Nb) exhibited lower furfural selectivity, likely due to enhanced secondary reactions, in contrast to the silica-supported catalysts. The SBA-15-supported catalyst with 12 wt% niobia (SBA-12Nb) achieved the highest performance, with 84% conversion and 93% furfural selectivity after 24 h at 160 °C in the biphasic system. Niobia loading had minimal impact on conversion, though 12 wt% provided optimal selectivity. Additionally, an alternative N_2_-stripping technique was explored, further improving furfural yield and product purity in the stripped stream.

Chareonlimkun et al. [54] investigated the simultaneous hydrolysis/dehydration reaction of sugarcane, rice husk, and corn cobs under hot compressed water conditions at 473–673 K in the presence of TiO_2_, ZrO_2_, and TiO_2_-ZrO_2_. Among them, the reaction of corncob at 573 K in the presence of TiO_2_–ZrO_2_ produced the highest furfural and 5-HMF yields (10.3% and 8.6%) with less by-product (i.e., glucose, fructose, xylose, and 1,6-anhydroglucose) selectivity.

Bakili et al. [50] reported that a SO_4_^2−^/TiO_2_–Nb_2_O_5_ (STNO) catalyst, prepared via a modified sol–gel method, exhibited excellent performance in the dehydration of xylose to furfural. The reaction was conducted in a biphasic solvent system of toluene and water. Under optimal conditions (150 °C, 180 min), the catalyst achieved a xylose conversion of 98 mol%, a furfural selectivity of 74 mol%, and a furfural yield of 63 mol%.

García-Sancho et al. [55] investigated the acid-catalyzed dehydration of D-xylose to furfural in a biphasic water–toluene system using a mesoporous Nb_2_O_5_ catalyst synthesized via a neutral templating method. The as-prepared mesoporous Nb_2_O_5_ exhibited significantly higher catalytic activity compared to commercial Nb_2_O_5_. Both D-xylose conversion and furfural yield increased with reaction temperature and time. At 170 °C and 90 min, the mesoporous catalyst achieved over 90% xylose conversion and more than 50% furfural yield.

Mishra et al. [56] employed Zn-doped CuO nanoparticles (NPs), synthesized via a sonochemical method, as catalysts for the dehydration of xylose to furfural. The catalytic performance of the Zn-doped CuO NPs was assessed using xylose as a model substrate and compared with that of ZnO NPs, ZnO bulk, CuO NPs, and CuO bulk materials. Owing to their high surface area, the Zn-doped CuO NPs exhibited superior catalytic activity, achieving complete conversion of xylose to furfural at 150 °C within 12 h. A furfural yield of up to 86 mol% was obtained, significantly surpassing the 45 mol% yield achieved with the other catalysts. Moreno-Marrodan et al. [57] developed catalysts based on a mixed niobium–titanium framework for the dehydration of xylose to furfural. The catalyst’s activity was found to depend on the niobium content in the titania lattice, with an increasing Lewis/Bronsted acid site ratio as the Nb content increased. The presence of as low as 2 wt% niobium resulted in the highest furfural yield of 42% at 140 °C under continuous-flow conditions, by using H_2_O/γ-valerolactone as a safe monophasic solvent system.

Rakngam et al. [58] developed an Al-SBA-15 catalyst by incorporating aluminum into SBA-15 using a hydrothermal method with different aluminum sources: sodium aluminate (SA), aluminum sulfate (AS), and aluminum isopropoxide (AI). SBA-15 is a stable mesoporous silica with a uniform pore structure but it lacks active catalytic sites. Introducing aluminum into the silica framework generated catalytic acid sites, enhancing the catalyst’s reactivity compared to the pure silica. Using the optimal catalyst Al-SBA-15(SA), a furfural yield of 63% was achieved at 170 °C after 7 h.

Xu et al. [59] reported that SBA-15 modified with phosphoric acid can effectively adsorb chromium ions (Cr^3+^), forming Cr^3+^/P-SBA-15 composites. These composites were tested as catalysts for the conversion of xylose and xylan into furfural. Among catalysts with varying chromium content, the (0.25)Cr^3+^/P-SBA-15 exhibited the highest activity, achieving furfural yields of 91% from xylose and 58% from xylan. Moreover, the catalyst demonstrated excellent stability, maintaining its performance after five reaction cycles.

Thus, the discussed catalytic materials showed different performances in the conversion of sugars to FUR (Table 1). The yield of furfural is influenced by the nature of the feedstock, type of catalyst, and process conditions for the production of FUR (solvent, temperature, and time). As the literature review demonstrates, research in recent years has focused on finding inexpensive and highly efficient solid acid catalysts and elucidating the complete reaction mechanisms for the sustainable conversion of biomolecules into high-value chemical products.

### 2.2. Production of 5-HMF and Levulinic Acid

**Figure 3:**
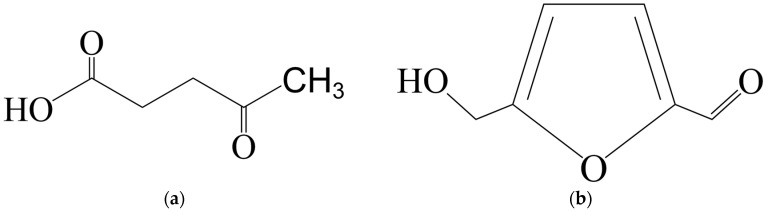
Structural formula of levulinic acid (**a**) and 5-HMF (**b**).

5-Hydroxymethylfurfural (5-HMF) and levulinic acid (LA) are the key chemical intermediates [60,61]. Levulinic acid (C_5_H_8_O_3_) is a carboxylic acid containing a keto group at the γ-position (γ-keto acid) produced by the degradation of C6-sugars or cellulose (Figure 3a). Due to the presence of both ketone and acid functional groups, LA is a highly reactive compound and readily undergoes hydrogenolysis, reduction, and cyclization. The 5-HMF molecule consists of a furan ring containing aldehyde and alcohol functional groups (Figure 3b).

LA is considered one of the top-12 platform molecules and is a building block for the synthesis of a wide range of products (hydrocarbon liquid fuels, solvents, pharmaceuticals, etc.) [61,62,63,64,65,66,67]. 5-HMF is an important target compound because it can be converted into a wide range of derivatives that can be used in the production of various products (solvents, polymers, resins, biofuels, etc.) [60,68,69]. The reviews [60,61,64,65,66,67,68] describe recent advances and challenges in the production of 5-HMF and levulinic acid.

**Figure 4:**
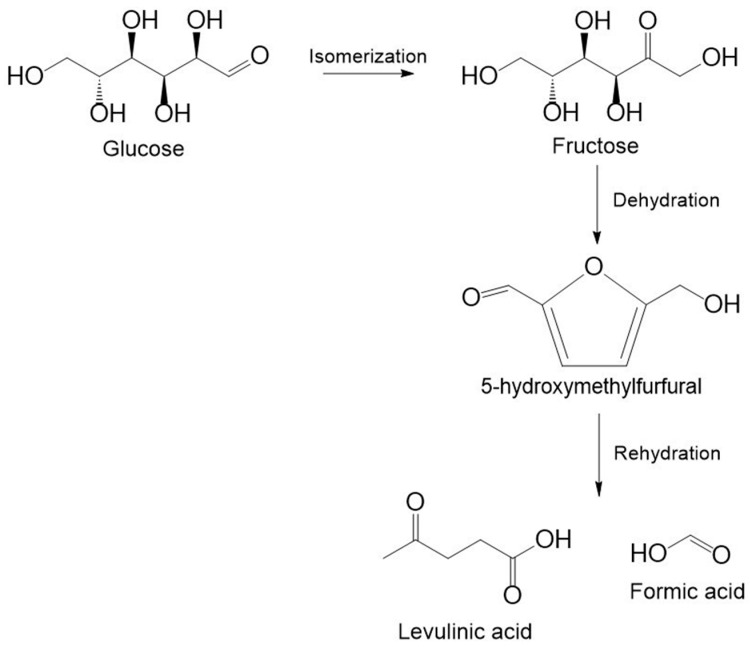
Production of 5-HMF and levulinic acid.

LA and 5-HMF can be produced from cellulosic biomass by acid-catalyzed reactions: the hydrolysis of cellulose to glucose, isomerization of glucose to fructose, dehydration of fructose to 5-HMF, and subsequent rehydration of 5-HMF to produce LA and formic acid (FA) simultaneously [60] (Figure 4). Bronsted and Lewis acids are mainly used in the hydrolysis and isomerization steps [67]. The following route for LA production is also possible: Pentose from hemicellulose is dehydrated to furfural, then furfural is converted to LA [69,70].

To obtain a high yield of 5-HMF by the dehydration of fructose in the presence of glucose, it is necessary to eliminate the decomposition of glucose to other by-products. The unconverted glucose is isomerized into fructose with further dehydration to 5-HMF [71]. Obtaining 5-HMF from a mixture of glucose and fructose is a cost-effective process [71,72]. Currently, research is still underway to establish efficient processes for the production of 5-HMF and levulinic acid. This section discusses the conditions for the preparation of 5-HMF and LA in the presence of different types of catalysts as well as their advantages and disadvantages.

Inorganic acids [71,73], metal salts [74,75], zeolites [76,77], ion exchange resins [78,79], phosphates [80,81], sulfonated solids [51,82], etc., are used as catalysts for the production of 5-HMF and levulinic acid. The industrial production of LA in homogeneous systems is problematic because of the need for a difficult purification stage and the problem of catalyst recycling [83].

Phosphates are recognized catalysts for the production of 5-HMF [80,81]. Hou et al. [80] tested a number of metal oxides and phosphates for 5-HMF production in ionic liquid. Tin phosphate gave high 5-HMF yields (58.3%). It was shown that tetracoordinated Sn^4+^ sites from tin phosphate are responsible for the isomerization of glucose into fructose. The conversion of fructose to 5-HMF is mainly catalyzed by the ionic liquid 1-ethyl-3-methylimidazolium bromide (EMIMBr). The excellent catalytic performance was attributed to the synergistic effect between tin phosphate and EMIMBr. Weingarten et al. [81] investigated zirconium and tin phosphate catalysts (ZrP, SnP) for producing LA from glucose via a two-step dehydration/rehydration process. Zirconium-based catalysts were synthesized with varying phosphorus-to-zirconium molar ratios: ZrP1 (P/Zr = 1), ZrP2 (P/Zr = 2), and ZrP3 (P/Zr = 3). ZrP catalysts exhibited higher total and Bronsted acid site concentrations compared to SnP. The catalytic performance was found to depend on the Bronsted/Lewis acid site ratio. ZrP2 and ZrP3 showed the highest 5-HMF selectivities—38% and 30%, respectively.

Souzanchi et al. [84] investigated niobium phosphate as a heterogeneous solid acid catalyst for the production of 5-HMF from industrial-quality sugar syrups derived from corn and wood. The yield of 5-HMF was 53.1% with 100% sugar conversion (glucose and fructose).

In study [85], the catalytic conversion of carbohydrates to 5-HMF was investigated using a heterogeneous zirconium phosphate catalyst doped with chromium (ZrP-Cr). Under optimized reaction conditions, fructose dehydration achieved a high 5-HMF yield of 94.5%, whereas glucose conversion resulted in a significantly lower yield of 43.2%.

Sulfonated SBA-15 is considered a promising catalyst support due to its high surface area, uniform mesoporous channels, and well-distributed pore sizes [82,86,87]. Cheng et al. [82] employed SBA-15 functionalized with propylsulfonic acid groups for the catalytic conversion of glucose to LA. Under optimal conditions (180 °C for 2.5 h. in a γ-valerolactone/water), the maximum LA yield was 61.56%.

Zeolite catalysts are widely recognized as effective in the production of platform chemicals, owing to their high acidity, thermal stability, and well-defined porous structure. A study [77] demonstrated that zeolites activated with sodium hydroxide solution provided LA yields of up to 42.7% from pentoses at 190 °C in 180 min. Wei et al. [88] synthesized Cr-modified HZSM-5 catalysts for the conversion of glucose to LA. The results showed that the catalyst with a larger surface area, high acid density, and suitable Lewis-Bronsted acid ratio was favorable for the production of LA from glucose. The highest LA yield of 64.4 mol% was achieved from glucose conversion in an aqueous medium at 180 °C for 180 min, using 0.75 g of 8%Cr/HZSM-5 catalyst. The activation energies of glucose dehydration and 5-HMF rehydration on the 8%Cr/HZSM-5 catalyst were 69.1 and 54.0 kJ/mol, respectively, which were significantly lower than those of HZSM-5. The impregnation of Cr in HZSM-5 enhances the Lewis acid of the catalyst, which ultimately accelerates the conversion of glucose through the isomerization of glucose to fructose, but the excess Lewis acid also leads to the conversion of glucose into by-products. The Cr/HZSM-5 catalyst showed good stability. The loss of activity was less than 13.1%.

Ramli et al. [89] developed an Fe/HY zeolite catalyst for the conversion of glucose. The main products in the temperature range from 120 to 200 °C were 5-HMF and LA. The highest yield of LA (66%) was obtained with 100% glucose conversion at 180 °C.

Zeolite SAPO-34 is a notable solid acid catalyst characterized by a high specific surface area and excellent hydrothermal stability. However, water generated during the dehydration reaction can block the zeolite’s acid sites, leading to a decline in catalytic activity [76]. To overcome this, Sun et al. [76] developed a hydrophobic version of SAPO-34 via a post-synthetic silanization method, grafting alkyl hydrophobic groups onto its surface. In the presence of SAPO-34 modified with dodecyltrimethoxysilane, the 5-HMF yield reached 95.05%. This surface modification enhances both the activity and selectivity of SAPO-34 by promoting selective water removal while maintaining efficient fructose conversion.

Recently, metal–organic frameworks (MOFs) composed of metal ions/clusters and organic linkers have attracted attention as heterogeneous catalysts due to their superior surface area, tunability, and inherent Lewis acidity [83]. Additional functional elements can be easily incorporated into MOF catalysts via pore encapsulation, metal chelation, and the modification of organic linkers [83]. Lee et al. [83] modified metal–organic framework UiO-66 with different types of Bronsted acid groups. The developed catalysts were investigated in the catalytic conversion of glucose to LA. In the presence of the UiO-66-NH-R-SO_3_H catalyst, the LA yield was 71.6%. Qu et al. [90] developed heterogeneous catalysts based on the metal–organic framework MIL-100 and iron (Fe) combined with insoluble lysine-functionalized phosphotungstic acid (Lys-PTA) for the conversion of glucose to LA in water. Using the prepared catalyst, the highest LA yield of 57.9% was achieved at 150 °C after 9 h.

Amberlyst-15 (A15), a sulfonated ion exchange resin, is widely used as a solid acid catalyst in the dehydration of fructose to 5-HMF [52,78,91]. Hu et al. [78] modified Amberlyst-15 with a cationic surfactant, cetyltrimethylammonium bromide (CTAB), through electrostatic interactions between the sulfonic acid groups of the resin and the quaternary ammonium cations of CTAB. This modification enhanced the surface hydrophobicity of the catalyst, thereby reducing humin adsorption and suppressing humin formation during fructose dehydration. As a result, the CTAB-modified Amberlyst-15 catalyst achieved 5-HMF yields ranging from 53.3 to 63.1 mol% in 1,4-dioxane/H_2_O solvent mixture at 140 °C for 2 h.

Dou et al. [92] reported the efficient production of 5-HMF via the catalytic dehydration of fructose using a novel catalyst composed of mesoporous silica functionalized with a perfluorosulfonic acid resin (Aquivion@silica). Data from physicochemical analysis methods indicate that the synthesized Aquivion@silica catalyst is characterized by large surface area and porosity and provides highly accessible sulfonic acid groups. The efficient and productive production of 5-HMF by fructose dehydration was achieved in the polar solvent dimethyl sulfoxide (DMSO) at a low temperature of 90 °C. The 5-HMF yield was 85%. Regeneration of the catalyst was carried out by a simple ion exchange method. After four times of use, the 5-HMF yield was stable. The authors [93] proposed a catalyst obtained by grafting active WO_3_ species onto a ZnCo_2_O_4_@CeO_2_ support for the efficient production of LA from corn cob biomass. The developed 4 wt% WO_3_/ZnCo_2_O_4_@CeO_2_ showed a good LA yield (78.5%) at 180 °C for 200 min. Its efficiency is attributed to its balanced acid–base surface stable over four cycles.

The Table 2 summarizes the results of studies on 5-HMF and LA production on different heterogeneous catalysts, including phosphate, oxide, zeolite, and sulfonated catalysts. Although certain catalysts can achieve high yields of LA and 5-HMF, limitations such as complex synthesis procedures, harsh reaction conditions, and poor catalyst stability often hinder their practical application. In comparison, heterogeneous catalysts that incorporate both Bronsted and Lewis acid sites within a single system have demonstrated superior performance. The synergistic effect of dual acidity enhances the catalytic conversion of carbohydrates to 5-HMF and LA, offering improved reaction efficiency and selectivity under milder conditions.

## 3. Hydrogenation of Platform Molecules Using Ru-Catalysts

Platform molecules such as 5-hydroxymethylfurfural (5-HMF)**,** levulinic acid (LA)**,** and furfural (FUR) are crucial intermediates in the deep processing of lignocellulosic biomass. These compounds contain reactive functional groups that offer numerous opportunities for chemical transformations aimed at producing a broad spectrum of high-value derivatives.

One of the key processes for further upgrading of 5-HMF, LA, and FUR is the hydrogenation of C=O bonds, which allows obtaining such value-added compounds as biofuels, solvents, pharmaceutical precursors, and monomers for polymers.

Ruthenium catalysts are known as the highly efficient systems for the selective hydrogenation of carbonyl compounds compared to other metals [94]. This enhanced performance makes Ru-based catalysts particularly promising for the hydrogenation of the lignocellulose-derived platform molecules.

### 3.1. Hydrogenation of Furfural

**Figure 5:**
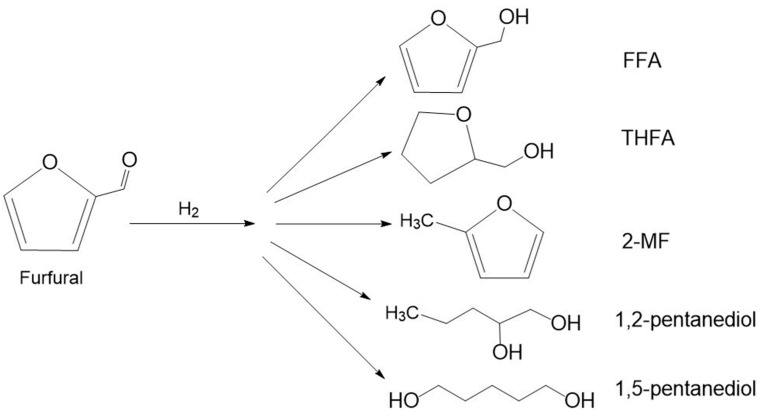
Furfural hydrogenation products.

Furfural is heterocyclic and aromatic aldehyde composed of a furan ring with an aldehyde side group [95]. The hydrogenation and hydrogenolysis of furfural allow to obtain a variety of valuable products. The most important products are furfuryl alcohol (FFA), tetrahydrofurfuryl alcohol (THFA), 2-methylfuran (2-MF); 1,2-pentanediol (1,2-PeD), and 1,5-pentanediol (1,5-PeD) (Figure 5).

#### 3.1.1. Furfuryl Alcohol

**Figure 6:**
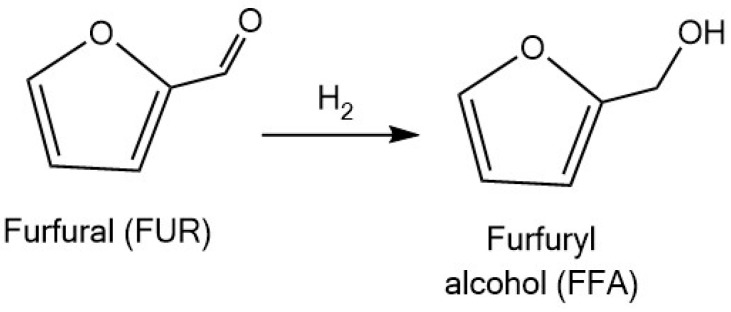
Hydrogenation of furfural to furfuryl alcohol.

Furfuryl alcohol (FFA) is one of the most important derivatives of furfural widely used in chemical industry. FFA is used in the production of resins, polyurethane foams, and polyesters. It also serves as a reactive solvent for phenolic resins, a viscosity reducer for epoxy resins, and a chemical building block for the synthesis of various pharmaceuticals [16]. It is typically obtained through the selective hydrogenation of the aldehyde functional group, while the furan ring is retained intact (Figure 6). The preservation of the furan ring during the selective hydrogenation of furfural to furfuryl alcohol requires strict control of reaction parameters, including temperature, pressure, and the nature of the catalyst [96].

Ru-based catalysts exhibit high activity and selectivity in the hydrogenation of furfural to furfuryl alcohol, particularly under liquid-phase conditions. Moreover, the efficiency and selectivity of the catalyst can be improved through the choice of an appropriate support material. For example, Kumaravel et al. [97] demonstrated that Al-modified mesoporous silica (Al-SBA-15) is excellent support for Ru catalysts. The 3.5 % Ru/Al-SBA-15, synthesized via a hydrothermal method, exhibited outstanding catalytic performance in the selective hydrogenation of furfural to furfuryl alcohol. Particularly, the catalyst achieved 100% conversion of furfural and 99% selectivity to furfuryl alcohol at ambient hydrogen pressure (1 atm) and temperature of 160 °C. Moreover, recycling experiments revealed that 3.5 % Ru/Al-SBA-15 can be reused at least five times without a significant change in conversion efficiency and product selectivity.

In [98], Ru nanoparticles supported on a series of zirconium-based metal–organic frameworks (UiO-66, UiO-67, Zr6-NDC, MIL-140A, MIL-140B, and MIL-140C) were evaluated in the selective hydrogenation of furfural to furfuryl alcohol under mild conditions (room temperature, 0.5 MPa H_2_). All catalysts showed promising catalytic performance with selectivity close to 100%. Among them, Ru/UiO-66 showed the highest catalytic activity, achieving a furfuryl alcohol yield of 94.9%. Additionally, this catalyst could be reused in five consecutive reaction cycles without a appreciable loss in performance.

Bardestani et al. [99] have reported that biochar with no surface area and low carboxyl groups surface density can be converted to an outstanding catalyst support using a very simple mild air/steam oxidation. Further impregnation of the mildly oxidized biochar with Ru(NH_3_)_6_Cl_2_ precursor resulted in the formation of a supported Ru catalyst with improved dispersion of active particles. As a result, the catalyst showed higher activity than the unoxidized one. Under optimized conditions (m_cat_ = 600 mg, T = 105 °C, P(H_2_) =1035 kPa, t = 25 h), this improved catalyst achieved a FFA selectivity of 93% at FUR conversion of 53%.

Tolek et al. [100] studied Ru-based catalysts supported on various TiO_2_ (anatase, rutile, P-25, and sol–gel TiO_2_) in the liquid-phase selective hydrogenation of furfural to FFA under mild conditions (50 °C and 2 MPa H_2_). The presence of a high-anatase crystallographic composition on the TiO_2_ support was found to be favorable for enhancing hydrogenation activity. Among the monometallic catalysts, the Ru catalyst supported on anatase (Ru/TiO_2_-A) demonstrated the highest activity, achieving a furfural conversion of 31.8%, and the selectivity to FFA was 90%. Further, the bimetallic Ru-Co/TiO_2_-A was prepared with different Co loading (0.2–0.8 wt%) at the same Ru content. The catalytic performances of bimetallic Ru–Co catalysts were improved with increasing Co loading. The bimetallic Ru-0.6Co/TiO_2_ catalyst showed the best catalytic performances for the selective hydrogenation of furfural to FFA with a 89.4% yield of FFA.

#### 3.1.2. Tetrahydrofurfuryl Alcohol

**Figure 7:**
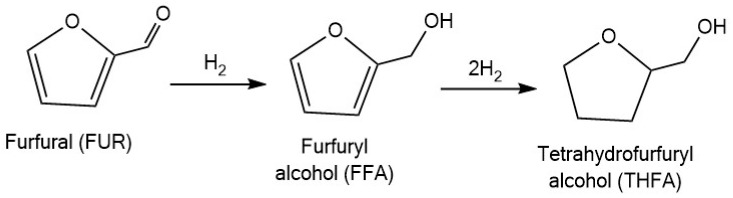
Simplified reaction pathway for the catalytic hydrogenation of FUR to THFA.

One of the most promising products derived from furfural processing is tetrahydrofurfuryl alcohol (THFA), which widely used as a green solvent in various industries, including agriculture and printing [101]. It also serves as a platform compound for the production of industrial resins, biofuel additives, printing inks, and electronic cleaners [101,102]. The production of THFA from furfural involves a sequential hydrogenation process, including the reduction of the aldehyde group followed by saturation of the furan ring (Figure 7). Ruthenium-based catalysts can be applied in this transformation after specific modifications or under alternative conditions (e.g., electrocatalytic hydrogenation).

Huang et al. [103] have reported that the complete hydrogenation of furfural to THFA can be achieved by using a combination of supported Pd and Ru catalysts. In particular, Pd/Al_2_O_3_ and Ru/ZrO_2_ mixture gave 99% yield of THFA at 30 °C and 0.5 MPa H_2_. Bruna et al. [104] demonstrated that in the hydrogenation of furfural, the selectivity of colloidal ruthenium nanoparticles stabilized with polyvinylpyrrolidone (Ru/PVP) can be tuned via in situ modification of the catalyst using various organic compounds. Specifically, the addition of hexadecylamine (HDA) to the reaction medium increased the selectivity toward THFA from 42% to 64%, and toward 1,2-pentanediol from 24% to 36%, at 125 °C under 20 bar of hydrogen pressure. More recently, electrocatalytic approaches have emerged as promising alternatives for furfural conversion under milder and greener conditions. Kasad et al. [105] investigated the electrocatalytic hydrogenation of furfural using a ruthenium catalyst supported on activated carbon cloth. Their study showed that the acidity of the catholyte solution had a significant impact on the yield of THFA. The highest THFA yield (48%) at 97% furfural conversion was achieved in mildly acidic catholyte solutions (0.02 M HCl and 0.002 M HCl–0.02 M NaCl) at 25 °C.

#### 3.1.3. 2-Methylfuran

**Figure 8:**
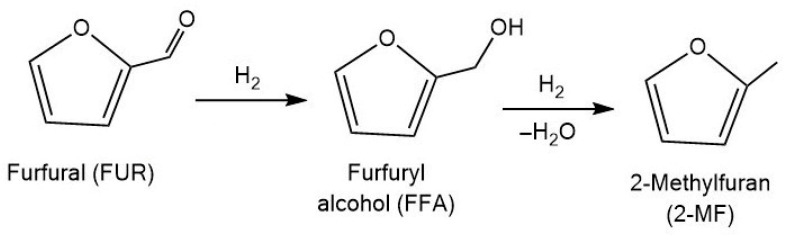
Simplified reaction pathway for the catalytic hydrogenation of FUR to 2-MF.

2-Methylfuran (2-MF) is a valuable biomass-derived compound with high solvent power, which is considered as promising biofuel component [106]. It also can be used for the synthesis of chrysanthemate pesticides, perfumes, and pharmaceutical intermediates [107]. The transformation of furfural to 2-MF involves the sequential reduction of the aldehyde group to a hydroxyl group, followed by hydrogenolysis of the C–O bond (Figure 8).

To achieve this conversion efficiently in a single step, ruthenium-based catalysts are often employed due to their high activity in the reduction of the aldehyde group. However, Ru alone typically lacks sufficient activity for the selective cleavage of the C–O bond. Therefore, the incorporation of a second metal into the catalytic system is generally required to enhance the hydrogenolysis step and improve overall selectivity toward 2-MF. For example, Aldosari et al. [108] demonstrated that the selectivity of bimetallic Pd-Ru/TiO_2_ catalysts for the selective hydrogenation of furfural to 2-MF can be finely tuned by varying the Pd to Ru ratio. The 1%Ru:4%Pd/TiO_2_ was found to be the most effective catalyst for this reaction, achieving the highest 2-MF yield (FUR conversion—39.3%, selectivity to 2-MF—51.5%) under mild reaction conditions (25 °C and 3 bar H_2_). In [109], Ru_x_/Ni_y_/SBA-16 (Ru_x_Ni_y_SB-16) catalysts with different weight percentages (x = 0.2, 0.4, 0.6, 0.8, 0.9 wt% and y = 0.8, 0.6, 0.4, 0.2, 0.1 wt%) were prepared through an in situ hydrothermal method and then studied in the catalytic hydrogenation of FUR to 2-MF. Among the catalysts tested, the Ru_0.8_Ni_0.2_SB-16 catalyst demonstrated outstanding performance under mild reaction conditions (180 °C, 1 atm), achieving 100% conversion of FUR and an impressive selectivity of 88% towards 2-MF. The high selectivity of 2-MF can be attributed to the combined influence of metal and acid sites on the catalyst surface. The catalyst remained reactive after five cycles with minimal loss, showing excellent stability. 

#### 3.1.4. Pentanediols

**Figure 9:**
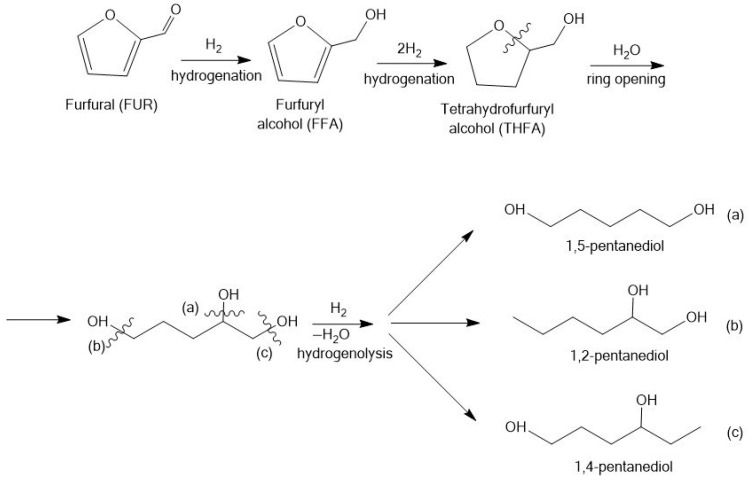
Reaction pathways for the catalytic transformation of FUR to various pentanediols.

Furfural can be transformed to various pentanediols such as 1,5-pentanediol (1,5-PeD), 1,2-pentanediol (1,2-PeD), and 1,4-pentanediol (1,4-PeD), which play crucial roles across multiple sectors. These include the synthesis of polymers and resins, as well as the production of diverse industrial products such as disinfectants, surfactants, printing inks, cosmetics, agrochemicals, and pharmaceuticals [110]. This transformation involves several key reactions, such as the reduction of the aldehyde group to a hydroxyl group, hydrogenation of the furan ring, ring-opening of the heterocycle, and hydrogenolysis of C–O bonds (Figure 9).

Recent studies have shown that this process can be carried out efficiently and selectively on bimetallic Ru–Sn catalysts. For example, Upare et al. [111] have reported that furfural can be selectively converted to 1,2-PeD with a very high yield (84.3%) in the presence of an Ru_3_Sn_7_ alloy catalyst supported on ZnO (Ru-Sn/ZnO) at 140 °C and 30 bar H_2_. The catalyst showed excellent catalytic performance and catalyst reusability when tested for up to five consecutive cycles without any significant deactivation. The remarkable performance of Ru-Sn/ZnO for the selective formation of 1,2-PeD from FUR was attributed to the combined effect of Ru_3_Sn_7_ alloy phases together with tin oxide species on a basic ZnO support. In contrast, Rodiansono et al. [112] used bimetallic a ruthenium–tin catalyst supported on gamma-alumina (Ru–Sn/γ-Al_2_O_3_) for the selective hydrogenolysis of furfural to 1,5-PeD. The presence of Ru_3_Sn_7_ alloy phases, Ru^0^, Sn^0^, and oxidative tin (SnO_x_) species on the sole surface of γ-Al_2_O_3_ was shown to synergistically catalyze the partial hydrogenation of C=C of FUR and hydrogenolysis of the C–O furan ring, thereby producing a high yield of 1,5-PeD (up to 94%) at 180 °C, under P(H_2_) = 30 bar and after reacting for 7 h.

Thus, ruthenium catalysts represent effective systems for the selective hydrogenation of furfural with the possibility of targeted production of various valuable products (Table 3).

In particular, Ru-supported catalysts are highly efficient in the selective hydrogenation of furfural to furfuryl alcohol. Furthermore, the catalytic properties of Ru-based systems can be finely tuned by incorporating a second metal or introducing organic ligands, allowing for enhanced selectivity toward other high-value products such as tetrahydrofurfuryl alcohol (THFA), 2-methylfuran (2-MF), and pentanediols. These transformations can be achieved with high selectivity and yield under optimized reaction conditions, including temperature, hydrogen pressure, and solvent choice.

### 3.2. Hydrogenation of Levulinic Acid

**Figure 10:**
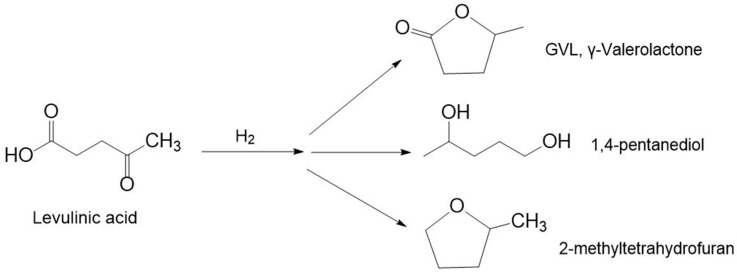
Levulinic acid hydrogenation products.

Levulinic acid (LA) is a gamma keto-acid, containing keto and carboxylic groups [113]. The hydrogenation and hydrogenolysis of these groups open up possibilities for further chemical transformations aimed at creating a wide range of valuable derivatives such as γ-valerolactone (GVL), 1,4-pentanediol (1,4-PeD), and 2-methyltetrahydrofuran (2-MTHF) (Figure 10).

#### 3.2.1. γ-Valerolactone

**Figure 11:**
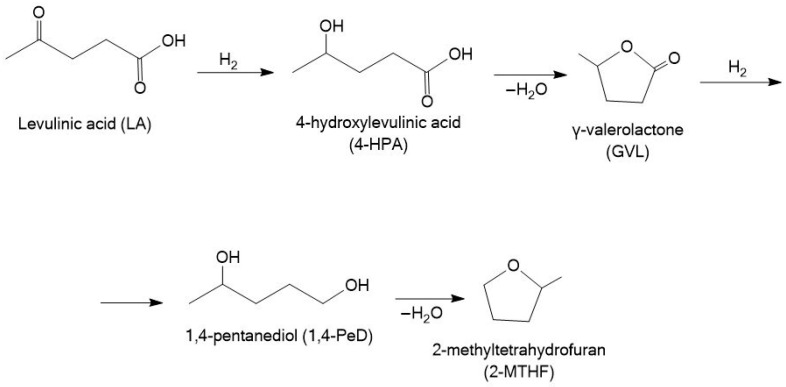
Reaction pathways for the catalytic transformation of LA to GVL, 1,4-PeD, and 2-MTHF.

The hydrogenation of levulinic acid (LA) to γ-valerolactone (GVL) is a key transformation in green and sustainable chemistry, offering a bridge between lignocellulosic biomass and valuable bio-based chemicals. GVL is a valuable intermediate compound, which can be used as a solvent, fuel, or raw material for fine chemical production [114]. This transformation includes the hydrogen reduction of the carbonyl group to obtain an intermediate 4-hydroxylevulinic acid (4-HPA) followed by intramolecular esterification (dehydration) to obtain GVL (Figure 11) [115].

The immobilization of Ru particles on solid supports with suitable acidity (Bronsted and/or Lewis’s acid sites) allows to obtain effective catalysts for this transformation. Piskun et al. [116] conducted a comprehensive screening study on the hydrogenation of LA to GVL in water using a wide range of ruthenium-supported catalysts in a batch set-up (1 wt% Ru, 90 °C, 45 bar of H_2_, 2 wt% catalyst on LA). Eight monometallic catalysts were tested on carbon based (C, carbon nanotubes (CNT)) and inorganic supports (Al_2_O_3_, SiO_2_, TiO_2_, ZrO_2_, Nb_2_O_5_, and Beta-12.5). The best result was found for Ru/Beta-12.5 with an almost quantitative LA conversion (94%) and 66% of GVL yield after 2 h reaction. Wang et al. [117] reported that the acidity of catalyst supports can be effectively tuned by preparing mixed metal oxides. In their study, Ru catalysts supported on Al_2_O_3_–TiO_2_, Al_2_O_3_–MoO_3_, and Al_2_O_3_–Co_3_O_4_ were tested in the hydrogenation of LA to GVL. The TiO_2_ component was found to significantly affect the acidity of the catalyst and, thus, its catalytic activity for the GVL yield was affected. The Ru/Al_2_O_3_–TiO_2_ catalyst achieved a GVL yield of approximately 97% under mild reaction conditions (WHSV = 1.8 h^−1^, T = 80 °C). Sorokina et al. [118] developed a Ru nanoparticle containing nanocomposites based on a hyperbranched pyridylphenylene polymer, serving as a multiligand and stabilizing matrix. The functionalization of the nanocomposite with sulfuric acid significantly enhances the activity of the catalyst in the selective hydrogenation of LA to GVL and allows the reaction to proceed under mild reaction conditions (100 °C, 2 MPa of H_2_) in water and low catalyst loading (0.016 mol.%) with a quantitative yield of GVL (99.9%) and selectivity up to 100%. The catalysts were successfully reused four times without a significant loss of activity. 

Jaya et al. [119] demonstrated that basic supports can also be employed to develop effective Ru-supported catalysts for the hydrogenation of LA to GVL under mild conditions. In their study, ruthenium supported on a magnesium–lanthanum mixed oxide (Ru/Mg–LaO) exhibited high catalytic activity at 80 °C and 0.5 MPa. The conversion of LA to GVL in toluene was found to be 92% with the selectivity > 99%. Moreover, the Ru/Mg–LaO catalyst exhibited excellent GVL yields for 5 cycles.

Several recent studies have shown that the preparation method and activation of ruthenium-based catalysts significantly influence the dispersion of Ru species, as well as the acid–base properties and overall catalytic efficiency. Rodríguez et al. [120] investigated the effect of the reduction temperature on the performance of a Ru catalyst supported on activated carbon for the hydrogenation of levulinic acid. Among the catalysts tested, the sample reduced at 200 °C exhibited the highest catalytic performance, achieving a GVL yield of 74% after 2 h at 70 °C and 1.5 MPa. This catalyst presented the lowest acidity value, and the ruthenium-containing phase consisted mainly of RuO_2_, with a small portion of Ru^0^. The catalyst demonstrated good reusability, maintaining a GVL yield of 56% after three consecutive reaction cycles. In [121], ruthenium-based catalysts were prepared through a deposition–precipitation approach, taking beta zeolites (BEA) with Si/Al ratios of 12.5, 18.5, and 150, respectively, as supports, and 1–3 wt% loadings of metal. The catalysts were also activated using either H_2_ or NaBH_4_. The catalysts reduced under H_2_ flow presented well-dispersed Ru^0^ and RuO_x_ nanoparticles, while the reduction with NaBH_4_ resulted in larger RuOₓ crystallites and highly dispersed Ru^0^ particles. The catalytic tests confirmed that the efficiency of Ru/BEA catalysts in LA hydrogenation is influenced by the activation protocol. However, the Ru loading and the Si/Al ratio of the zeolite support affected the LA conversion more significantly. Regardless of the Si/Al ratio, increasing the Ru loading from 1 to 3 wt% led to an increase in activity. Also, for the catalysts with the same loading of ruthenium, an increase in the conversion of LA was determined by increasing the Si/Al ratio from 12.5 to 150. An optimal combination of these features was achieved for the catalyst with 3 wt% Ru and a Si/Al ratio of 150, which reached 96.5% LA conversion and 97.8% selectivity to GVL at 130 °C and 10 bars of H_2_.

#### 3.2.2. 1,4-Pentanediol (1,4-PeD)

1,4-pentanediol (1,4-PeD) plays a pivotal role in the synthesis of polyether polyols, polyester resins, coatings, adhesives, and polyurethane elastomers, showcasing its versatility across various industrial products and processes [110]. It can be also used as an intermediate for dyes and of pharmaceuticals and pesticides, and a precursor for the production of various heterocyclic compounds (e.g., 1-methylpiperidine) [122]. This compound can be obtained from both furfural and levulinic acid. The transformation of LA to 1,4-PeD typically involves several sequential steps: the hydrogenation of the keto group in LA to form 4-hydroxypentanoic acid (4-HPA) and the cyclization of 4-HPA to produce γ-valerolactone (GVL), followed by the hydrogenation and hydrogenolysis of GVL with a lactone ring opening to yield 1,4-pentanediol (Figure 11) [122]. Ruthenium-based catalysts with appropriate acid–base properties are particularly effective for facilitating this multistep transformation.

Lv et al. [123] demonstrated that nanoporous Ru prepared by the leaching of Al from RuAl alloy exhibited attractive catalytic activity for LA hydrogenation to 1,4-PeD using H_2_O as solvent. The total yield of 1,4-PeD was 74.6% acquired in 140 °C and 100 °C. This catalyst outperformed conventional Ru-based catalysts (such as powder Ru, 5% Ru/C, and 5% Ru/Al_2_O_3_) in both activity and selectivity, owing to its unique acid sites and specific lattice planes, which effectively promoted the activation of the lactone group of GVL.

In [124], an efficient and green process was developed for the direct conversion of levulinic acid into 1,4-pentanediol over a Mo-modified Ru/activated carbon (AC) catalyst in a continuous fixed-bed reactor. Compared with the unmodified Ru/AC catalyst, the Ru–MoO_x_/AC catalyst displayed much higher activity and selectivity at low temperature (70 °C), achieving of a 1,4-pentanediol yield of 96.7%. The high efficiency of this catalyst can be attributed to the combined effect of the closely connected Ru and Mo species. The Ru nanoparticles were used to activate and dissociate H_2_, whereas Mo species with oxygen vacancies can act as Lewis’s acid sites and are capable of interacting with the lone electron pair of oxygen in the carbonyl group (C=O), by which the C=O bonds in GVL intermediate are weakened and finally hydrogenated to 1,4-PeD.

Lee et al. [125] have reported that the combination of ruthenium (Ru), a hydrogenating metal, and rhenium (Re), an oxophilic promoter, results in high activity for 1,4-PeD production from LA. In their study, bimetallic RuRe nanoparticles with various atomic ratios and monometallic Ru and Re were supported on various carbon materials with different surface properties such as activated carbon (AC), carbon black (CB), mesoporous carbon (CMK-3), and carbon nanofiber (CNF). These catalysts were characterized using a range of physicochemical techniques and evaluated in the hydrogenation of LA. The catalytic activity and 1,4-PeD selectivity were found to be correlated with the metal particle size, pore structure, and surface oxygen functionalities of the catalysts. Notably, smaller RuRe nanoparticles with a Re-enriched surface supported on carbon with a larger pore size exhibited a higher 1,4-PeD production rate. Among the catalysts, RuRe supported on CB exhibited the highest 1,4-PeD selectivity (~75%) with LA conversion of 99% at 130 °C and 50 bar of H_2_.

#### 3.2.3. 2-Methyltetrahydrofuran

2-Methyltetrahydrofuran (2-MTHF) is a commercially available solvent derived from biomass feedstocks such as levulinic acid and furfural. Its physical and chemical properties, such as its low miscibility with water, boiling point, apolar aprotic chemical characteristic, abiotic degradability, and remarkable acid and basic stability compared to other cyclic-based solvents, such as tetrahydrofuran, make it appealing for multiple applications [126]. The conversion of LA to 2-MTHF involves a multi-step reaction pathway: initial hydrogenation of LA to GVL, followed by further hydrogenation to 1,4-PeD, and finally, cyclodehydration of 1,4-PeD to yield 2-MTHF (Figure 11) [122,127]. Similar to the synthesis of 1,4-PeD, the conversion of LA to 2-MTHF can proceed efficiently over ruthenium-based catalysts in the presence of acidic sites.

Phanopoulos et al. [128] evaluated a series of pre- or in situ-formed ruthenium complexes with branched triphosphine ligands for the stepwise catalytic hydrogenation of LA to 2-MTHF via GVL and 1,4-PeD. Reactions were conducted at 150 °C, under 65 bar of H_2_ pressure for 25 h. The most active catalyst was the preformed ruthenium species [RuH_2_(PPh_3_){N(CH_2_PPh_2_)_3_-κ^3^*P*}], which gave a near-quantitative conversion of LA to 1,4-PeD when no acidic additives were present, and a 87% yield of 2-MTHF when used in conjunction with HN(Tf)_2_. Various acidic additives were assessed to promote the final transformation of 1,4-PeD to 2-MTHF. However, only HN(Tf)_2_ was found to be effective, which likely due to the noncoordinating nature of the acid, which will not compete with the substrate for binding to the metal center.

Licursi et al. [129] investigated the catalytic hydrogenation of LA with the objective of selectively producing either 2-MTHF or mono-alcohols (2-butanol and 2-pentanol) as final products. Under optimized conditions (180 °C, 5.0 MPa of H_2_, 3 h), a combination of 5 wt% Ru/C (10 mg) and 10 wt% Re/C (20 mg) together with niobium phosphate (NBP, 500 mg) as an acid co-catalyst led to increasing the yield of 2-MTHF up to ~28 mol%.

In the work by Upare et al. [130], Ru-based catalysts supported on activated carbon (Ru/C) and graphene oxide (Ru/GO) were evaluated for the hydrogenation of LA in a fixed-bed reactor. It was found that using GO as a support material led to significantly improving the selectivity of the Ru catalyst towards cyclic ethers (2-MTHF and tetrahydrofuran-THF) compared to the Ru/C catalyst. The 5% Ru/GO catalyst successfully produced 53.5% of cyclic ethers (48% 2-MTHF and 5.5% THF) under moderate reaction conditions (265 °C, 25 bar of H_2_, WHSV—0.512 h^−1^; time—50 h). To improve the yield of cyclic ethers, a two-step hydrogenation strategy was applied. In this approach, GVL obtained from LA hydrogenation was further hydrogenated over Ru/GO under using reaction conditions similar to those discussed for LA hydrogenation. The 5% Ru/GO catalyst successfully produced 91% of cyclic ethers (69% MTHF and 16% THF) and 15% of other hydrocarbons from intermediate GVL at 100% conversion. Thus, Ru/GO catalysts showed a 92% selectivity of predominantly cyclic ethers, including a 77% selectivity of MTHF through a two-step process. Such a remarkable enhancement in the activity and selectivity of LA hydrogenation over Ru/GO can be attributed to the better dispersion of metallic nanoparticles of Ru on GO than on carbon, stronger metal support interaction, and higher acidity due to the presence of functional groups (-carboxyl, -epoxy, -hydroxyl, etc.) on the GO surface.

Thus, the hydrogenation of levulinic acid using ruthenium catalysts is a highly efficient route to obtaining a number of valuable products (Table 4).

The development of effective Ru-based catalysts for the selective transformation of LA into GVL, 1,4-PeD, and 2-MTHF requires careful tuning of their acid–base properties. This can be achieved by selecting solid supports with suitable acidity (zeolites, Al_2_O_3_, TiO_2_, and oxidized carbon materials), modifying supported Ru catalysts with oxophilic promoters (MoO_x_ and Re), and their activation with H_2_ at specific temperature. The yield of desired products can also be improved by using a combination of Ru catalysts with an acid co-catalyst and optimizing the reaction conditions.

### 3.3. Hydrogenation of 5-Hydroxymethylfurfural

**Figure 12:**
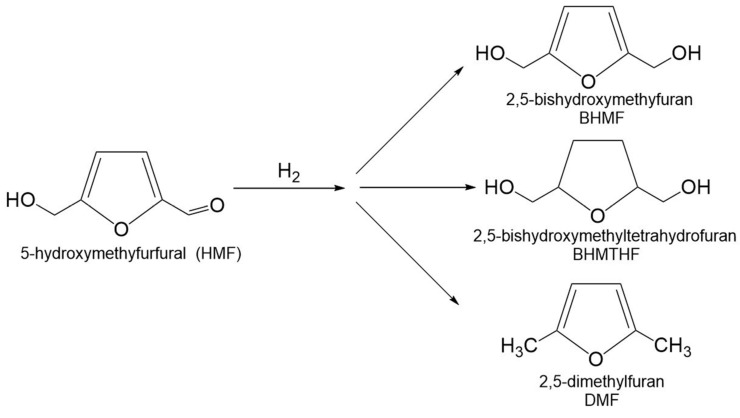
5-Hydroxymethyfrfural hydrogenation products.

5-Hydroxymethylfurfural (5-HMF) is a multifunctional molecule because it is at the same time an aromatic aldehyde, an aromatic alcohol, and a furan ring system [131]. The hydrogenation of 5-HMF is key process in converting biomass into a variety of valuable chemical products such as 2,5-Bis(hydroxymethyl)furan (BHMF), 2,5-Bis(hydroxymethyl)tetrahydrofuran (BHMTHF), 2,5-dimethylfuran (DMF), etc. (Figure 12).

#### 3.3.1. 2,5-Bis(hydroxymethyl)furan and 2,5-Bis(hydroxymethyl)tetrahydrofuran

**Figure 13:**
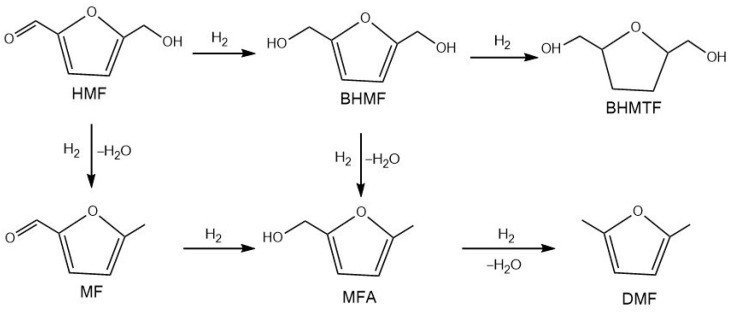
Reaction pathways for the catalytic hydrogenation/hydrogenolysis of 5-HMF to BHMF, BHMTH,F and DMF.

2,5-Bishydroxymethylfuran (BHMF) is a key intermediate obtained via mild hydrogenation of the aldehyde group in 5-hydroxymethylfurfural (5-HMF). Further hydrogenation of furan ring of BHMF lead to the formation of 2,5-bis(hydroxymethyl)tetrahydrofuran (BHMTHF) (Figure 13). Both the diols play an important role in the production of biopolymers (polyurethanes and polyesters) and serve as versatile building blocks for the synthesis of pharmaceuticals and biofuel additives [132,133,134]. Ruthenium catalysts supported on solid materials are widely used in the hydrogenation of 5-HMF to either BHMF or BHMTHF, owing to their high activity and selectivity under mild reaction conditions.

Jain and Vaidya [135] investigated the reaction kinetics of 5-HMF hydrogenation in aqueous solution using the 5%Ru/C catalyst in a slurry reactor. Their study demonstrated that 5-HMF is selectively hydrogenated to BHMF with 100% selectivity at lower temperatures in the range of 40–70 °C. However, increasing the temperature beyond 100 °C resulted in the conversion of 5-HMF and BHMF into 2,5-bis-hydroxymethyltetrahydrofuran (BHMTHF), 5-methylfurfuryl alcohol (MFA), and 2,5-dimethylfuran (DMF).

A similar 5% Ru/C catalyst has been employed for the hydrogenation and hydrogenolysis of 5-HMF under both single-phase (aqueous) and batch multiphase (MP) conditions [136]. By changing reaction parameters, experiments were optimized towards the formation of three products: 2,5-bis(hydroxy methyl)furan (BHMF), 2,5-bis(hydroxymethyl)tetrahydrofuran (BHMTHF), and 1-hydroxyhexane-2,5-dione (HHD). In particular, the single-phase (aqueous) hydrogenation of 5-HMF, conducted at 60 °C under 30 bar of H_2_ for 18 h, enabled the exclusive formation of BHMF with 92% selectivity at a quantitative conversion of 85%. The hydrogenation/hydrogenolysis of 5-HMF was also investigated under continuous-flow conditions using various solvents, including ethyl acetate, tetrahydrofuran, and ethanol. At 100 °C, 50 bar of H_2_, and a flow rate of 0.1 mL min^−1^, the process was optimized towards the formation of the full hydrogenation product BHMTHF, achieving 100% 5-HMF conversion with 90% selectivity. Ethyl acetate proved the best solvent.

Fulignati et al. [137] investigated the hydrogenation of aqueous 5-HMF solutions (2–3 wt%) using three commercial catalysts, Ru/C, Pd/C, and Pt/C, with a metal loading of 1 wt% relative to the 5-HMF content. By appropriate tuning of the process conditions, either BHMF or BHMTHF was obtained in a good yield, and Ru/C was the best catalyst for this purpose. Specifically, the highest BHMTHF yield of 95.3 mol% was achieved from a 3 wt% 5-HMF aqueous solution at 100 °C and 50 bar of H_2_ after 240 min. In contrast, under milder conditions (50 °C, 30 bar of H_2_, 240 min), the BHMF yield reached a maximum of 93.0 mol%.

In [138], the catalytic transfer hydrogenation (CTH) of 5-HMF to BHMF was investigated using isopropanol as the hydrogen donor and Ru/Co_3_O_4_ as the catalyst. The study demonstrated that Ru/Co_3_O_4_ exhibited high catalytic efficiency, achieving a BHMF yield of up to 82% at 190 °C after 6 h. Furthermore, the recovered Ru/Co_3_O_4_ catalyst retained a catalytic performance comparable to that of the fresh catalyst, indicating good stability and reusability.

Kashyap et al. [139] investigated Ru/TiO_2_ catalysts in the hydrogenation of 5-HMF, conducted at 120 °C under 70 bar of H_2_ for 6 h. Their study demonstrated that the performance and selectivity of Ru/TiO_2_ toward valuable furan-based diols (BHMF and BHMTHF) can be finely tuned by adjusting the H_2_ pre-treatment temperature of the catalyst. In particular, increasing the reduction temperature from 200 °C to 400 °C led to a gradual increase in both the 5-HMF conversion and the BHMF yield, from 68% up to 100% for the 5-HMF conversion, and from 61% up to 98% for the BHMF yield. This enhancement was attributed to the optimized formation of Ru–TiOₓ interfacial sites at 400 °C, which balances metal–support interactions while preventing excessive TiO_x_ coverage of Ru nanoparticles. In addition, extending the reaction time from 6 h to 24 h on the optimal Ru/TiO_2_ catalyst reduced at 400 °C, led to a progressive switch of the reaction selectivity from BHMF to BHMTHF while maintaining a total 5-HMF conversion, so that the yield to BHMTHF reached 100% after 24 h of reaction.

Mishra et al. [140] also demonstrated the possibility of the selective production of either BHMF or BHMTHF from the hydrogenation of 5-HMF by simply varying the reaction time. In their study, Ru/MnCo_2_O_4_ was found to be the most effective catalyst. Under optimized conditions (100 °C, 8.2 MPa of H_2_), the catalyst achieved a BHMF yield of 98.5% at complete 5-HMF conversion (100%), and a BHMTHF yield of 97.3% at a slightly lower 5-HMF conversion (98.7%). Specifically, BHMF was obtained after 4 h at a 5-HMF/Ru molar ratio of 100, whereas BHMTHF was formed after 16 h at a 5-HMF/Ru ratio of 50. These excellent results were attributed to the presence of Bronsted acid sites (10.7 mmol/g) on the surface of MnCo_2_O_4_ spinels support and enhanced Lewis acidity (7.3 mmol/g), resulting from the incorporation of Ru nanoparticles.

#### 3.3.2. 2,5-Dimethylfuran

2,5-dimethylfuran (DMF) derived from 5-HMF is considered as promising green alternative fuel to gasoline. It has ideal fuel properties such as a high octane number (119), which is higher than those of gasoline and ethanol, high boiling point (92–94 °C), and low water solubility (0.26%) [141]. DMF also serves as an intermediate for the production of terephthalate polymers [142]. The transformation of 5-HMF into DMF can be achieved by hydrogenation/hydrogenolysis, in which hydrogen or hydrogen-donor participates and removes the oxygen as water. The process initiates with the hydrogenation of the carbonyl group in 5-HMF to yield BHMF. This is followed by hydrogenolysis, which cleaves the –C–OH bonds in BHMF, ultimately yielding DMF (Figure 13) [143].

Ruthenium supported on various carriers exhibits high activity in the conversion of 5-HMF to DMF. In a representative study, Hu et al. [144] evaluated a series of metal catalysts (Raney-Ni, Pd/C, Pt/C, Rh/C, and Ru/C) in the selective hydrogenation of 5-HMF into DMF using THF as reaction medium. Among the employed metal catalysts, Ru/C displayed the highest catalytic performance, which led to a 94.7% DMF yield with 100% 5-HMF conversion at a relatively mild reaction condition (200 °C, 20 bar of H_2_) for only 2 h. Moreover, Ru/C exhibited good catalytic stability. It could be recycled at least six times after a simple regeneration process by heating at a mixed flow of H_2_ and N_2_.

Dong et al. [145] reported that the introduction of a second metal (Co) and optimization of the reduction protocol can significantly enhance the catalytic properties of Ru-based systems for the hydrogenation/hydrogenolysis of 5-HMF to DMF. Their study focused on Ru–Co bimetallic catalysts supported on activated carbon (AC), systematically examining the effects of Ru/Co ratios, Ru loading, and reduction strategies on catalytic performance. The addition of cobalt was found to improve both the dispersion of Ru nanoparticles on the AC support and facilitate electron transfer from Co to Ru, thereby enhancing the catalytic activity. Ru NPs act as an active hydrogenation site and CoOx serves as the acid site to synergistically activate and crack the C–O bonds during the hydrogenolysis reaction of 5-HMF. In addition, it was found that the highest DMF yield of 5%Ru–1%Co/AC could be achieved by first reducing Ru by NaBH_4_ followed by impregnating with Co, and, finally, reducing it by H_2_. An excellent yield of DMF up to 97.9% and 98.7% conversion of 5-HMF was achieved in a short time (1.5 h) under the optimal conditions (200 °C, 1.0 MPa). Moreover, the catalyst has good recyclability and could be reused at least three times without a significant loss in activity, and the slight deactivation was attributed to the increase in particle sizes and the leaching of the active metal.

Another compelling example of synergistic interaction between Ru particles and CoOx species is reported in a study involving a Ru/Co_3_O_4_ catalyst, prepared by a simple co-precipitation method [146]. This catalyst exhibited excellent catalytic performance in the conversion of 5-HMF into DMF, achieving a DMF yield of 93.4% at relatively low reaction temperature and H_2_ pressure (130 °C, 0.7 MPa) for 24 h. Additionally, the catalyst also displayed a good reusability and can be used five times without loss of the activity.

Buta et al. [147] developed a ruthenium catalyst supported on nitrogen-doped ordered mesoporous carbon (Ru/N-CMK-1), which exhibited high performance for the selective conversion of 5-HMF to DMF via catalytic transfer hydrogenolysis using isopropanol as a hydrogen donor. The nitrogen-promoted carbon support enhanced the dispersion of Ru due to the formation of appropriate basic site density which could efficiently promote the activation of alcohol hydroxyl in isopropanol and subsequent release of active hydrogen species. In the meantime, highly dispersed surface Ru nanoparticles were beneficial for hydrogen transfer and activation of both carbonyl and hydroxyl groups in 5-HMF. As a result, a complete 5-HMF conversion with a high DMF yield of 88% was achieved under optimized reaction conditions (160 °C, 20 bar of N_2_, 8 h). Additionally, the catalyst demonstrated excellent stability without obvious loss of activity after three consecutive cycles, which was explained by superior metal–support interaction and the mesoporous framework nature of the catalyst.

Thus, ruthenium-based catalysts have been extensively employed for the valorization of 5-HMF into a range of value-added products, including BHMF, BHMTHF, and DMF (Table 5).

Ruthenium particles supported on carbon materials or metal oxides have been shown to be effective catalysts for the transformation of 5-HMF into BHMF under mild reaction conditions. By increasing the reaction temperature and prolonging the reaction time, the selectivity of these catalysts can be tuned toward the formation of more deeply hydrogenated products such as BHMTHF and DMF.

## 4. Ru-Catalyzed One-Pot Conversion of Carbohydrates into Value Added Products

Modern biorefining technologies aim to simplify biomass conversion pathways as much as possible, minimizing the number of processing steps while reducing both energy and resource consumption [4]. Typically, the transformation of carbohydrates into value-added products is a multistep process, involving the initial synthesis of platform compounds such as 5-hydroxymethylfurfural, levulinic acid, and furfural, which are subsequently converted into target products via hydrogenation or hydrogenolysis. A wide range of solid acid catalysts have been employed for the transformation of carbohydrates into platform molecules such as furfural (FUR), levulinic acid (LA), and 5-hydroxymethylfurfural (5-HMF) (Table 1 and Table 2). These include acidified carbon materials, zeolites (e.g., SAPO-34, H-Beta, ZSM-5), and modified mesoporous silicas (such as Al-SBA-15 and Cr^3+^/P-SBA-15), as well as various metal oxides and phosphates (e.g., Al_2_O_3_, Nb_2_O_5_, ZrO_2_, TiO_2_, SnPO, NbPO, ZrPO). Notably, some of these materials have also been employed as supports in the development of ruthenium-based catalysts for the subsequent hydrogenation or hydrogenolysis of the platform compounds (Table 3, Table 4 and Table 5). This observation suggests that such bifunctional metal–acid catalysts hold significant potential for the one-pot conversion of carbohydrates into valuable chemicals, with platform molecules acting as key intermediates within the integrated catalytic process. This assumption is supported by the work of Insyani et al. [148]. In their study, bifunctional RuO_2_–Ru catalysts supported on Hβ zeolite (RuO_2_–Ru/Hβ) were used for the one-pot cascade conversion of xylose to tetrahydrofurfuryl alcohol (THFA). The process involved the consecutive isomerization and dehydration of xylose to FUR and subsequent hydrogenation of FUR to THFA. To establish structure–property relationships and optimize THFA yield through sequential isomerization, dehydration, and hydrogenation, various catalyst preparation methods were explored, including incipient wetness impregnation, reductive deposition, activated reductive deposition, and post-oxidative activated reductive deposition (ARD-O). The best performance was achieved with the RuO_2_–Ru/Hβ-ARD-O catalyst, which delivered nearly complete xylose conversion and a high THFA yield of 61.8% after 1 h at 180 °C under 3.0 MPa of H_2_ in THF, with the feed to catalyst ratio of 5 (*w*/*w*).

Another approach for the direct conversion of carbohydrates involves the use of Ru-based catalysts in combination with solid acid co-catalysts. In [149], the one-pot conversion of xylose to 1,2-pentanediol was investigated in a dual catalyst system composed of Ru/C and niobium phosphate as hydrogenation and acid catalysts, respectively. The study found that the ratio of Lewis to Bronsted acid sites in the niobium phosphate significantly influenced the product distribution. Catalysts with a higher proportion of Lewis acid sites favored the formation of 1,2-pentanediol and 1-hydroxy-2-pentanone, while suppressing the formation of xylitol. At a Lewis-to-Bronsted acid site ratio of 2.5, a selectivity of 14.0% for 1,2-pentanediol and 13.3% for 1-hydroxy-2-pentanone was achieved at complete xylose conversion under moderate reaction conditions (150 °C, 3.0 MPa H_2_, 4 h, 16.0 g of water, 16.0 g of cyclohexane, 0.2 g of xylose, 200 mg of acid catalyst, 50 mg of hydrogenation catalyst). Ren et al. [150] proposed a promising one-pot approach for the catalytic production of GVL from carbohydrates (fructose, glucose, starch, and cellulose) using a combination of 1-methyl-3-(3-sulfopropylimidazolium)silicotungstate ([MIMPS]_4_SiW) as a Bronsted acid catalyst and Ru/ZrO_2_ as a hydrogenation catalyst. A 63% yield of GVL with 99% conversion of fructose (0.5 g) was obtained using 10 mol% of [MIMPS]_4_SiW and 0.1 g of Ru/ZrO_2_ at 180 °C in pure water. The reaction proceeded in two steps: an initial 3 h dehydration under 0.1 MPa of N_2_, followed by a 10 h hydrogenation under 4 MPa of H_2_. Under the same reaction conditions, the GVL yields from glucose, starch, and cellulose were 68%, 60%, and 60%, respectively. The recyclability of the catalytic system consisting of [MIMPS]_4_SiW and Ru/ZrO_2_ was investigated in four runs. The activity for [MIMPS]_4_SiW did not change after use. Partial deactivation of Ru/ZrO_2_ was observed, which was ascribed to the severe carbon deposition. Duan et al. [151] reported a one-step conversion of fructose, glucose, and polysaccharides composed of fructose or glucose into tetrahydro-2,5-furandimethanol (THFDM) using a dual catalyst system consisting of niobic acid treated with HF and H_3_PO_4_ (Nb_2_O_5_-FP) and a methyl-functionalized ruthenium catalyst (Ru/SiO_2_-TM). The Nb_2_O_5_-FP was proved to have medium and strong acid sites with a high Brönsted/Lewis ratio, which played a large role in maintaining high THFDM selectivity using glucose as a substrate. The glucose (1 mmol) conversion reached 49%, with a THFDM selectivity of approximately 60% under reaction conditions of 160 °C, 4 MPa H_2_, 30 mg of Ru/SiO_2_-TM, 40 mg of Nb_2_O_5_-FP, and a reaction time of 8 h. The dual catalytic system can be reused for at least four runs without significant loss in activity and selectivity.

Thus, ruthenium-based catalysts in combination with acidic co-catalysts are capable of directly converting carbohydrates into value-added products such as GVL, THFA, THFDM, and 1,2-pentanediol. Although the yields of target products from direct carbohydrate conversion (10–60%) are generally lower compared to those achieved via the hydrogenation or hydrogenolysis of platform molecules (up to 100%), the integration of dehydration and subsequent hydrogenation/hydrogenolysis steps in a single reactor is a promising strategy, as it eliminates the need for complex purification and separation of intermediates. As demonstrated by recent studies, the acid–base properties of the support in bifunctional catalysts or of the co-catalyst in dual catalytic systems play a crucial role in ensuring the selective formation of target compounds both from carbohydrates and from platform molecules. Therefore, the approaches used in designing highly efficient catalysts for the hydrogenation of platform compounds (selecting supports with appropriate acid–base characteristics, adjusting the amount of acidic co-catalyst, optimizing the reduction conditions of the ruthenium catalyst, introducing oxophilic promoters, and fine-tuning reaction parameters) can also be effectively applied to enhance the yields of desired products from carbohydrates.

## 5. Conclusions

The catalytic conversion of biomass-derived carbohydrates into high-value-added chemicals and fuels is one of the most important topics in the field of sustainable chemistry and the recycling of renewable resources. This review was focused on the acid-catalyzed transformation of carbohydrates into platform molecules such as 5-hydroxymethylfurfural, levulinic acid, and furfural, followed by their selective hydrogenation/hydrogenolysis using ruthenium-based catalysts to produce valuable downstream products.

Literature analysis reveals notable progress in the development of effective solid acid catalysts for the transformation of carbohydrates into platform molecules. Various classes of solid catalysts such as carbon- and silica-based materials, zeolites, and metal oxides, or phosphates incorporating elements like Sn, Al, Nb, Cu, Ti, Zr, and Ce have demonstrated promising activity. Furthermore, the performance of these catalysts can be enhanced through modifications with acidic functional groups or transition metals such as Cr, Zn, W, and Fe. These advancements enable the production of key platform molecules with good-to-high yields (40–96%) under relatively mild reaction conditions (140–190 °C).

Furfural, 5-hydroxymethylfurfural, and levulinic acid contain reactive carbonyl groups (aldehyde or ketone), the hydrogenation of which serves as a key step in the synthesis of a broad spectrum of value-added compounds—ranging from solvents and biofuels to pharmaceutical agents, agrochemicals, cosmetic ingredients, and polymers. Ruthenium-based catalysts exhibit high efficiency in the selective hydrogenation of carbonyl groups to the corresponding alcohols, making them highly attractive for the further valorization of these platform molecules. However, in addition to the selective reduction of the carbonyl group (C=O), these processes are accompanied by several side reactions, such as hydrogenation of the furan ring, hydrogenolysis of C–O bonds, opening of the heterocyclic ring, and intramolecular esterification.

Therefore, to enhance the selectivity and steer the pathways of the deep conversion of platform compounds, ruthenium catalysts are subjected to targeted modification. The main approaches involve tuning the acid–base properties of the catalyst surface and controlling the dispersion of ruthenium particles. This can be achieved by doping with oxophilic promoters (e.g., Re, Mo, Co), surface modification with acidic groups or organic ligands, selecting suitable supports containing Lewis and/or Bronsted acid sites, and optimizing reductive activation protocols to obtain the desired Ru/RuO_x_ species ratio. As a result, such catalysts can deliver high yields of target products (up to 100%) under optimized reaction conditions. Another effective way to increase the yield of high-value-added products is a combination of ruthenium-based catalysts with other materials serving as co-catalysts. Notably, in both approaches, the catalytic systems incorporate components that have proven to be efficient solid acid catalysts for the conversion of carbohydrates into platform molecules. This opens up the possibility of integrating consecutive biomass conversion steps (namely, the transformation of carbohydrates into platform molecules, followed by their hydrogenation/hydrogenolysis) into a single process. Indeed, although studies in this area remain limited, existing data demonstrate the potential of bifunctional metal–acid catalysts and dual catalytic systems based on ruthenium for the direct conversion of carbohydrates.

From the perspective of green chemistry, the direct conversion of carbohydrates represents a promising approach to biomass valorization compared to multi-step cascade processes, as it reduces energy consumption and eliminates the need for the complex purification and separation of intermediate products. Further research in this area should focus on the development of ruthenium-based catalysts that ensure higher yields, improved selectivity, and long-term stability. In our view, this can be achieved by identifying an optimal balance between Lewis and Bronsted acid sites and the metallic centers of ruthenium, in order to fine-tune activity and selectivity for specific reactions. To this end, several complementary approaches can be employed:

- Development of advanced supports with tailored acid–base properties and high surface area, enhancing Ru dispersion, catalytic activity, and resistance to deactivation;
- Design and synthesis of bifunctional Ru-based catalysts that integrate hydrogenation and acid-catalyzed functionalities within a single material for efficient one-pot carbohydrate conversion;
- Optimization of catalyst preparation conditions to control the size, morphology, and dispersion of Ru nanoparticles;
- Development of bi- and multi-metallic systems, including doping of Ru nanoparticles with oxophilic promoters (e.g., Re, Mo, W) or additional metals such as Pd, to improve hydrogenation activity, guide selectivity, and suppress side reactions;
- Surface modification by acidic groups (Bronsted centers), organic ligands, or transition-metal compounds (to introduce or strengthen Lewis acidity);
- Adjustment of catalyst pretreatment protocols to achieve the desired Ru^0^/RuOx ratio, which directly influences activity and selectivity.

In addition, the use of real lignocellulosic feedstocks and the optimization of reaction conditions are essential to ensure the practical applicability and scalability of these catalytic systems under industrially relevant conditions.

Ultimately, the goal in this field is to develop robust, selective, and scalable catalytic platforms for the direct transformation of renewable biomass into a diverse portfolio of sustainable chemicals and fuels. Achieving this will contribute significantly to the global transition toward a circular and carbon-neutral bioeconomy, replacing fossil-based feedstocks with renewable resources in a cost-effective and environmentally friendly manner.

## Figures and Tables

**Table 1:** The heterogeneous catalysts for furfural production.

Catalyst	Reaction Conditions	Furfural Yield, %	Ref.
Carbon based catalysts			
S-800-CG	T = 140 °C t = 40 min m_cat_ = 100 mg solvent—1,4-dioxane xylose—200 mg	85.9	[33]
SC-GCa-800	T = 140 °C t = 40 min m_cat_ = 50 mg solvent—1,4-dioxane xylose—100 mg	76.9	[34]
ZnCS-2	T = 180 °C t = 60 min m_cat_ = 5 mg solvent—methyl isobutyl ketone /NaCl xylose—100 mg	72.4	[35]
MCSA	T = 190 °C t = 120 min m_cat_ = 5 mg solvent—H_2_O/γ-valerolactone tobacco stalks—60 mg	46.6	[39]
EAC-3H-2T	T = 180 °C t = 3 h m_cat_ = 1.5 wt% solvent—γ-valerolactone xylose—100 mg	74.6	[41]
SG	T = 150 °C t = 40 min m_cat_ = 28 mg solvent—γ-valerolactone xylose—60 mg	96.0	[42]
Zeolite based catalysts			
BEATUD	T = 170 °C t = 8 h m_cat_ = 20 mg solvent—H_2_O D-xylose—30 mg	74.0	[29]
ZSM-5	T = 463 K t = 3 h m_cat_ = 1.0 g solvent—organic solvent/aqueous waste aqueous hemicellulose solution—15 mL	82.4	[30]
SAPO-34	T = 463 K t = 8 h m_cat_= 48 mg solvent—γ-valerolactone/H_2_O xylose or switchgrass—2 wt%	40.0	[43]
H-Beta	T = 160 °C t = 40 min m_cat_= 150 mg solvent—H_2_O monosaccharide—2 wt%	73.0	[44]
Cr-deAl-Y	T = 180 °C t = 30 min solvent—H_2_O/n-butanol ratio catalyst/xylose = 2:4 (*w*/*w*)	77.5	[45]
HZ-Na-50	T = 150 °C t = 60 min m_cat_ = 20 mg solvent—H_2_O/toluene xylose—625 mg	63.0	[46]
HSO_3_-ZSM-5	T = 160 °C t = 5 h m_cat_ = 20 mg solvent—THF/H_2_O corncob—20 mg	89.0	[47]
Metal oxide-based catalysts			
γ-Al_2_O_3_	T = 175 °C t = 30 min m_cat_ = 50 mg solvent—H_2_O/toluene xylose—150 mg	83.0	[52]
Mesoporous niobia (Nb450)	T = 170 °C; t = 90 min m_cat_ = 50 mg D-xylose—150 mg solvent—H_2_O/toluene	50.0	[55]
Zn doped CuO	T = 150 °C t = 12 h m_cat_ = 100 mg ratio cat/xylose = 1:5 (*w*/*w*) solvent—H_2_O	86.0	[56]
NbTiO-MNL2	T = 140 °C t = 141s m_cat_ = 114 mg xylose—0.02 M solvent—H_2_O/γ –valerolactone	42.0	[57]
Al-SBA-15(SA)	T = 170 °C t = 7 h m_cat_ = 100 mg solvent—H_2_O xylose—0.9 g	63.0	[58]
Cr^3+^/P-SBA-15	T = 170 °C t = 90 min m_cat_ = 30 mg xylose—100 mg solvent—H_2_O/THF	91.0	[59]

**Table 2:** The different catalysts for 5-HMF and LA production.

Catalyst	Reaction Conditions	Yield 5-HMF/ LA, %	Ref.
5-HMF production			
SnPO	T = 120 °C t = 3 h The catalyst/glucose ratio = 1:2 (*w/w*) solvent—ionic liquid EMIMBr	58.3	[80]
ZrP-Cr	T = 120 °C t = 2 h m_cat_ = 100 mg fructose—180 mg solvent—1-butyl-3-methylimidazolium chloride	94.5	[85]
CTAB-modified Amberlyst-15	T = 140 °C t = 2 h solvent—1,4-dioxane/H_2_O	63.1	[78]
NbPO_4_	T = 150 °C corn syrup—200 mg/mL aqueous to organic phase ratio of 1:5 (*v/v*).	53.1	[84]
Fe/HY zeolite	T = 120 °C t = 60 min solvent—H_2_O glucose—5000 ppm	11.4	[89]
Aquivion@silica	T = 90 °C t = 2 h solvent—DMSO fructose—300 mg catalyst—16 μmol H^+^	85.0	[92]
LA production			
AZ25	T = 190 °C t = 180 min ratio C5 sugar/zeolite = 2:1 (*w*/*w*) solvent—H_2_O	42.7	[77]
CH_3_-SBA-15-SO_3_H	T = 180 °C t = 2.5 h ratio catalyst/glucose = 5:1 (*w*/*w*) solvent—GVL/H_2_O	61.56	[82]
UiO-66-NH-R-SO_3_H	T = 170 °C solvent—H_2_O	71.6	[83]
8%Cr/HZSM-5	T = 180 °C t = 180 min m_cat_ = 0.75 g solvent—H_2_O	64.4	[88]
Fe/HY zeolite	T = 180 °C t = 240 min solvent—H_2_O glucose–5000 ppm	66.0	[89]
WO_3_/ZnCo_2_O_4_@CeO	T = 180 °C t = 200 min catalyst dosage—4 wt% corncob biomass—5 g solvent—H_2_O	78.5	[93]

**Table 3:** Hydrogenation of furfural to various valuable products.

Product	Catalyst	Reaction Conditions	Results	Reference
Furfuryl alcohol (FFA)	3.5% Ru/Al-SBA-15	T = 160 °C P(H_2_) = 1 atm m_cat_ = 150 mg t = 4 h	FUR conversion—100%, selectivity to FFA—99%	[97]
	Ru/UiO-66	T = 20°C P(H_2_) = 5 bar t = 4 h m_cat_ = 100 mg FUR—10 µL solvent—H_2_O	yield of FFA—94.9%	[98]
	Ru/C (mildly oxidized biochar)	T = 105 °C P(H_2_) = 1035 kPa t = 25 h m_cat_ = 400 mg FUR—1 g	FUR conversion—53%, selectivity to FFA—93%	[99]
	Ru-Co/TiO_2_ (0.6%Co)	T = 50 °C P(H_2_) = 2 MPa t = 2 h m_cat_ = 50 mg FUR—50 µL solvent—methanol	FUR conversion—91.7%, selectivity to FFA—97.5%	[100]
Tetrahydro-furfuryl alcohol (THFA)	Pd/Al_2_O_3_ (60 mg) + Ru/ZrO_2_ (100 mg)	T = 30 °C P(H_2_) = 0.5 MPa t = 3 h FUR—0.1 mL solvent—H_2_O	yield of THFA—99%	[103]
	Ru/PVP + HDA	T = 125 °C P(H_2_) = 20 bar t = 48 h m_cat_ = 10 mg FUR—4 mmol solvent—1-propanol	yield of THFA—64%, yield of 1,2-PeD—36%	[104]
	Ru/C (electrocatalysis)	T = 25 °C solvent—mildly acidic catholyte solutions (0.02 M HCl) 100 mA t = 2 h	FUR conversion—97%, yield of THFA—48%	[105]
2-Methylfuran (2-MF)	1%Ru:4%Pd/TiO_2_	T = 25 °C P(H_2_) = 3 bar t = 3 h m_cat_ = 100 mg FUR—1 g solvent—octane	FUR conversion—39.3%, selectivity to 2-MF—51.5%	[108]
	Ru_0_._8_Ni_0_._2_/SBA-16	T = 180 °C P(H_2_) = 1 atm t = 4 h m_cat_ = 200 mg FUR—1mL solvent—2-propanol	FUR conversion—100%, selectivity to 2-MF—88%	[109]
Pentanediols	Ru-Sn/ZnO (Ru_3_Sn_7_)	T = 140 °C P(H_2_) = 30 bar t = 6 h m_cat_ = 100 mg	1,2-PeD—84.3%	[111]
	Ru-SnO_x_/γ-Al_2_O_3_	T = 180 °C P(H_2_) = 30 bar t = 7 h m_cat_ = 50 mg FUR—2.0 mmol solvent—1,4-dioxane	yield of 1,5-PeD—94%	[112]

**Table 4:** Hydrogenation of levulinic acid to various valuable products.

Product	Catalyst	Reaction Conditions	Results	Ref.
GVL	Ru/Beta-12.5	T = 90 °C P(H_2_) = 45 bar t = 2 h m_cat_ = 60 mg LA—0.6–0.7 mol L^−1^	LA conversion—94%, yield of GVL—66%	[116]
	Ru/Al_2_O_3_-TiO_2_	T = 80 °C WHSV = 1.8 h^−1^ P(H_2_) = 4.0 MPa m_cat_ = 2g LA—5 wt% solvent—H_2_O	yield of GVL—97%	[117]
	Ru@HBPPS/SO_3_H	T = 100 °C P(H_2_) = 2.0 MPa t = 4h catalyst loading—0.016 mol.% LA—1g solvent—H_2_O	yield of GVL—99.9%, selectivity to GVL—100%	[118]
	Ru/Mg-LaO	T = 80 °C P(H_2_) = 0.5 MPa t = 4h m_cat_ = 100 mg LA—2g solvent—toluene	LA conversion—92%, selectivity to GVL > 99%	[119]
	Ru/AC (reduced at 200 °C)	T = 70 °C P(H_2_) = 1.5 MPa t = 2 h m_cat_ = 100 mg LA—2.3g solvent—H_2_O	yield of GVL—74%	[120]
	Ru/BEA (3 mass.%, Si/Al = 150)	T = 130 °C P(H_2_) = 10 bar m_cat_ = 10 mg t = 24 h LA—1 mmol solvent—1,4-dioxane	LA conversion—96.5%, selectivity to GVL—97.8%	[121]
1,4-PeD	Nanoporous Ru	T = 120 °C P(H_2_) = 6 MPa t = 24 h m_cat_ = 10 mg LA—2 mmol solvent—H_2_O	yield 1,4-PeD—74.6%	[123]
	Ru–MoOx/AC	T = 70 °C P(H_2_) = 4 MPa WHSV—0.4h^−1^ m_cat_ = 4.3 g LA—5 wt% solvent—H_2_O	yield of 1,4-PeD—96.7%	[124]
	RuRe/CB	T = 130 °C P(H_2_) = 50 bar	LA conversion—99%, selectivity to 1,4-PeD ~ 75%	[125]
2-MeTHF	[RuH_2_(PPh_3_){N(CH_2_PPh_2_)_3_}] + HN(Tf)_2_	T = 150 °C P(H_2_) = 65 bar t = 25 h catalyst—0.5 mol% LA—10 mmol solvent—THF	yield of 2-MeTHF—87%	[128]
	Ru/C (10 mg) + Re/C (20 mg) + NBP (500 mg)	T = 180 °C P(H_2_) = 5 MPa t = 3 h LA—1.99 g solvent—H_2_O	yield of 2-MeTHF ~ 28 mol.%	[129]
	Ru/GO	T = 265 °C P(H_2_) = 25 bar WHSV—0.512 h^−1^ t = 50 h m_cat_ = 1.0 g solvent—1,4-dioxane	selectivity to ether 92%, to 2-MeTHF—77%	[130]

**Table 5:** Hydrogenation of 5- HMF to various valuable products.

Product	Catalyst	Reaction Conditions	Results	Ref.
BHMF	5% Ru/C	T = 40–70 °C P(H_2_) = 0.69–2.07 MPa t = 1 h 5-HMF—19.8–39.7 mM catalyst loading—0.3–0.7 kg m^–3^ aqueous phase	5-HMF conversion—31–95%, selectivity to BHMF—100%	[135]
	Ru/C	T = 60 °C P(H_2_) = 30 bar t = 6 h m_cat_ = 50 mg 5-HMF—0.2 M aqueous phase	5-HMF conversion—85%, selectivity to BHMF—92%	[136]
	Ru/C	T = 50 °C P(H_2_) = 30 bar t = 240 min Ru/5-HMF ratio = 1 wt% 3 wt% 5-HMF aqueous solution	BHMF yield—93.0 mol%	[137]
	Ru/Co_3_O_4_	T = 190 °C t = 6 h 5-HMF—0.5 wt% catalyst loading—0.25 wt% catalytic transfer hydrogenation, isopropanol as the hydrogen donor	5-HMF conversion—100%, yield of BHMF—82.8%	[138]
	Ru/TiO_2_ reduced at 400 °C	T = 120 °C P(H_2_) = 70 bar t = 6 h m_cat_ = 150 mg 5-HMF—1 g solvent—1,4-dioxane	BHMF yield—98%	[139]
	Ru/MnCo_2_O_4_	T = 100 °C P(H_2_) = 8.2 MPa 5-HMF/Ru =100 t = 4 h	HMF conversion—100%, BHMF yield —98.5%	[140]
BHMTHF	Ru/C	T = 100 °C P(H_2_) = 50 bar run time—60 min flow rate = 0.1 mL min^−1^ m_cat_ = 300 mg 0.05 M 5-HMF in ethyl acetate	5-HMF conversion—100%, selectivity to BHMTHF—90%	[136]
	Ru/C	T = 100 °C P(H_2_) = 50 bar t = 240 min Ru/5-HMF ratio = 1 wt% 3 wt% 5-HMF aqueous solution	BHMTHF yield—95.3%	[137]
	Ru/TiO_2_ reduced at 400 °C	T = 120 °C P(H_2_) = 70 bar t = 24 h m_cat_ = 150 mg 5-HMF—1 g solvent—1,4-dioxane	BHMTHF yield—100%	[139]
	Ru/MnCo_2_O_4_	T = 100 °C P(H_2_) = 8.2 MPa 5-HMF/Ru = 50 t = 16 h	5-HMF conversion—98.7%, yield of BHMTHF—97.3%	[140]
DMF	Ru/C	T = 200 °C P(H_2_) = 2 MPa t = 2 h catalyst—5mol% 5-HMF—2.5 wt% solvent—THF	5-HMF conversion—100%, yield of DMF—94.7%	[144]
	Ru–Co/AC (5% Ru, 1% Co)	T = 200 °C P(H_2_) = 1.0 MPa t = 1.5 h m_cat_ = 25 mg 5-HMF—1.25 wt% solvent—THF	5-HMF conversion—98.7%, yield of DMF—97.9%	[145]
	Ru/Co_3_O_4_	T = 130 °C P(H_2_) = 0.7 MPa t = 24 h m_cat_ = 100 mg 5-HMF—0.25 g solvent—THF	yield of DMF—93.4%	[146]
	2% Ru/N-CMK-1	T = 160 °C P(N_2_) = 20 bar t = 8 h m_cat_ = 10 mg 5-HMF—20mM catalytic transfer hydrogenolys isopropanol as a hydrogen donor	yield of DMF—88%	[147]

## Data Availability

No new data were created or analyzed in this study. Data sharing is not applicable to this article.

## Abbreviations

The following abbreviations are used in this manuscript:

1,2-PeD	1,2-pentanediol
1,4-PeD	1,4-pentanediol
1,5-PeD	1,5-pentanediol
2-MF	2-methylfuran
2-MTHF	2-methyltetrahydrofuran
4-HPA	4-hydroxylevulinic acid
5-HMF	5-hydroxymethylfurfural
AC	Activated carbon
AI	Aluminum isopropoxide
ARD-O	Post-oxidative activated reductive deposition
AS	Aluminum sulfate
BEA	Beta zeolites
BEATUD	Beta zeolite nanocrystals embedded in silicon mesoporous matrix
BHMF	2,5-Bis(hydroxymethyl)furan
BHMTHF	2,5-Bis(hydroxymethyl)tetrahydrofuran
CB	Carbon black
CMK-3	Mesoporous carbon
CNF	Carbon nanofiber
CNT	Carbon nanotubes
CTAB	Cetyltrimethylammonium bromide
DMF	2,5-dimethylfuran
DMSO	Dimethyl sulfoxide
EAC	Eucalyptus-derived activated carbon
EMIMBr	ionic liquid 1-ethyl-3-methylimidazolium bromide
FA	Formic acid
FFA	Furfuryl alcohol
FUR	Furfural
GVL	γ-valerolactone
HDA	Hexadecylamine
HHD	1-hydroxyhexane-2,5-dione
LA	Levulinic acid
LC	Crude lignin
Lys-PTA	Lysine-functionalized phosphotungstic acid
MCSA	Magnetic carbon-based solid acid
MFA	5-methylfurfuryl alcohol
[MIMPS]_4_SiW	1-methyl-3-(3-sulfopropylimidazolium)silicotungstate
MOF	Metal-organic framework
NBP	Niobium phosphate
SA	Sodium aluminate
THF	Tetrahydrofuran
THFA	Tetrahydrofurfuryl alcohol
THFDM	Tetrahydro-2,5-furandimethanol
TsOH	p-toluenesulfonic acid
WHSV	Weight hourly space velocity

## References

[1] Du X., Zhang J., Wang Y., Qu Y. (2017). Conversion of Carbohydrates into Platform Chemicals Catalyzed by Alkaline Ionic Liquids. Catalysts.

[2] Vaithyanathan V.K., Goyette B., Rajagopal R. (2023). A critical review of the transformation of biomass into commodity chemicals: Prominence of pretreatments. Environ. Chall..

[3] Hommes A., Heeres H.J., Yue J. (2019). Catalytic Transformation of Biomass Derivatives to Value-Added Chemicals and Fuels in Continuous Flow Microreactors. ChemCatChem.

[4] Dutta S. (2024). Catalytic Transformation of Carbohydrates into Renewable Organic Chemicals by Revering the Principles of Green Chemistry. ACS Omega.

[5] da Silva M.J., Rodrigues A.A., Batalha D.C. (2024). Furfural and Levulinic Acid: Synthesis of Platform Molecules from Keggin Heteropolyacid-Catalyzed Biomass Conversion Reactions. Reactions.

[6] Esteban J., Yustos P., Ladero M. (2018). Catalytic Processes from Biomass-Derived Hexoses and Pentoses: A Recent Literature Overview. Catalysts.

[7] Zhang Y., Wang S., Yang Y., Wang L., Xu E., Hou Q., Zhao S., Liu T., Hong S., Zheng L. (2024). A switchable hydrogenation chemoselectivity of biomass platform compounds based on solvent regulation. Appl. Catal. B: Environ. Energy.

[8] Xia H., Xu S., Hu H., An J., Li C. (2018). Efficient conversion of 5-hydroxymethylfurfural to high-value chemicals by chemo- and bio-catalysis. RSC Adv..

[9] Li X., Zhang L., Wang S., Wu Y. (2020). Recent Advances in Aqueous-Phase Catalytic Conversions of Biomass Platform Chemicals Over Heterogeneous Catalysts. Front. Chem..

[10] Zhang K., Wang J., Tian Y., Zhang S., Chen S.S., Cao L., Zhang J., Clark J.H., Zhang S. (2025). Ruthenium catalysts for hydrogenation of biomass-based levulinic acid for efficient γ-valerolactone synthesis. iScience.

[11] Seretis A., Diamantopoulou P., Thanou I., Tzevelekidis P., Fakas C., Lilas P., Papadogianakis G. (2020). Recent Advances in Ruthenium-Catalyzed Hydrogenation Reactions of Renewable Biomass-Derived Levulinic Acid in Aqueous Media. Front. Chem..

[12] Akram M., Bhutto S.U.A., Aftab S., Wang F., Xu X., Xia M. (2023). Ruthenium based with carbon supported catalysts for the catalytic transfer hydrogenation of furfural: A review. Nano Energy.

[13] Gebresillase M.N., Errol D.S., Jeong G.S. (2024). Interplay Between Metallicity and Acidity in the Hydrogenation of Levulinic Acid. ACS Sustain. Chem. Eng..

[14] Liu M., Liu Y. (2024). Highly efficient hydrogenation of levulinic acid into γ-valerolactone over modified bifunctional yolkshell catalyst. J. Phys. Conf. SeriesJ. Phys. Conf. Ser..

[15] Yu I.K.M., Tsang D.C.W. (2025). Insights into the kinetics of furfural production from different monomers and polymers derived from biomass in a subcritial water reaction medium intensified by CO_2_ as pressurization agent. Biomass Bioenergy.

[16] Sener C., Motagamwala A.H., Alonso D.M., Dumesic J.A. (2018). Enhanced Furfural Yields from Xylose Dehydration in the γ-Valerolactone/Water Solvent System at Elevated Temperatures. ChemSusChem.

[17] Kabbour M., Luque R., Saravanamurugan S., Pandey A., Li H., Riisager A. (2020). Furfural as a platform chemical: From production to applications. Biomass, Biofuels, Biochemicals Recent Advances in Development of Platform Chemicals.

[18] Mariscal R., Maireles-Torres P., Ojeda M., Sádaba I., López Granados M. (2016). Furfural: A renewable and versatile platform molecule for the synthesis of chemicals and fuels. Energy Environ. Sci..

[19] Edumujeze D., Fournier-Salaün M.-C., Leveneur S. (2025). Production of furfural: From kinetics to process assessment. Fuel.

[20] Gan P., Zhang K., Yang G., Li J., Zhao Y., Chen J. (2024). Catalytic Production and Upgrading of Furfural: A Platform Compound. Int. J. Mol. Sci..

[21] Wang Y., Li M., Wang Z., Liu S., O’Young L. (2025). Furfural production: A review on reaction mechanism and conventional production process. Ind. Crops Prod..

[22] Illera A.E., Candela H., Bermejo-López A., Barea P., Alonso-Riaño P., Benito-Román Ó., Beltrán S., Sanz M.T. (2024). Evaluation of homogeneous and heterogeneous catalytic strategies for furfural production from sugar-derived biomass in a solvent-free green pressurized reaction media (subcritical water-CO_2_). Biomass Bioenergy.

[23] Choudhary V., Sandler S.I., Vlachos D.G. (2012). Conversion of xylose to furfural using Lewis and Brønsted acid catalysts in aqueous media. ACS Catal..

[24] Tongtummachat T., Jaree A., Akkarawatkhoosith N. (2022). Continuous hydrothermal furfural production from xylose in a microreactor with dual-acid catalysts. RSC Adv..

[25] Lamminpää K., Ahola J., Tanskanen J. (2015). Acid-catalysed xylose dehydration into furfural in the presence of Kraft lignin. Bioresour. Technol..

[26] Delbecq F., Wang Y., Muralidhara A., El Ouardi K.E., Marlair G., Len C. (2018). Hydrolysis of hemicellulose and derivatives-a review of recent advances in the production of furfural. Front. Chem..

[27] Zhang L., Yu H., Wang P., Dong H., Peng X. (2013). Conversion of xylan, D-xylose and lignocellulosic biomass into furfural using AlCl_3_ as catalyst in ionic liquid. Bioresour. Technol..

[28] Yang T., Zhou Y.H., Zhu S.Z., Pan H., Huang Y.B. (2017). Insight into Aluminum Sulfate-Catalyzed Xylan Conversion into Furfural in a γ-Valerolactone/Water Biphasic Solvent under Microwave Conditions. ChemSusChem.

[29] Lima S., Antunes M.M., Fernandes A., Pillinger M., Ribeiro M.F., Valente A.A. (2010). Catalytic cyclodehydration of xylose to furfural in the presence of zeolite H-Beta and a micro/mesoporous Beta/TUD-1 composite material. Appl. Catal. A Gen..

[30] Gao H., Liu H., Pang B., Yu G., Du J., Zhang Y., Wang H., Mu X. (2014). Production of furfural from waste aqueous hemicellulose solution of hardwood over ZSM-5 zeolite. Bioresour. Technol..

[31] Xu S., Pan D., Wu Y., Song X., Gao L., Li W., Das L., Xiao G. (2018). Efficient production of furfural from xylose and wheat straw by bifunctional chromium phosphate catalyst in biphasic systems. Fuel Process. Technol..

[32] Cheng L., Guo X., Song C., Yu G., Cui Y., Xue N., Peng L., Guo X., Ding W. (2013). High performance mesoporous zirconium phosphate for dehydration of xylose to furfural in aqueous-phase. RSC Adv..

[33] Xu Z., Zhang G., Wang K. (2023). Efficient conversion of biomass derivatives to furfural with a novel carbon-based solid acid catalyst. Catal. Commun..

[34] Yang T., Li W., Su M., Liu Y., Liu M. (2020). Production of furfural from xylose catalyzed by a novel calcium gluconate derived carbon solid acid in 1,4-dioxane. New J. Chem..

[35] Zhou S., Zhang X., Zhan L., Song X., Li R., Wu Y. (2025). Future development for furfural production: Comparison of one-step and two-step strategies and life cycle assessment. Ind. Crops Prod..

[36] Li W., Zhu Y., Lu Y., Liu Q., Guan S., Chang H., Jameel H., Ma L. (2017). Enhanced furfural production from raw corn stover employing a novel heterogeneous acid catalyst. Bioresour. Technol..

[37] Qiu B., Shi J., Hu W., Wang Y., Zhang D., Chu H. (2024). Efficient and selective conversion of xylose to furfural over carbon-based solid acid catalyst in water-γ-valerolactone. Energy.

[38] Antonyraj C.A., Haridas A. (2018). A lignin-derived sulphated carbon for acid catalyzed transformations of bio-derived sugars. Catal. Commun..

[39] Zhang Z., Lu L., Xu H., Lu X., Li X. (2025). A magnetic carbon-based solid acid catalyst derived from tobacco stalk for efficient valorization of tobacco stalk to furfural. Ind. Crops Prod..

[40] Liu Y., Lin Q., Zhao L., Zhu R., Wang X., Ren J., Qi W., Li L. (2024). Mechanism insights into the upgrading of xylose to furfural over the carbon-based catalysts in aqueous media. Fuel.

[41] Yusuff A.S., Gu Y. (2024). Studies on effective catalytic conversion of xylose to furfural using green sulfonated carbon catalysts: Process optimization by Taguchi approach. Arab. J. Chem..

[42] Ma J., Li W., Guan S., Liu Q., Li Q., Zhu C., Yang T., Ogunbiyi A.T., Ma L. (2019). Efficient catalytic conversion of corn stalk and xylose into furfural over sulfonated graphene in γ-valerolactone. RSC Adv..

[43] Bruce S.M., Zong Z., Chatzidimitriou A., Avci L.E., Bond J.Q., Carreon M.A., Wettstein S.G. (2016). Small pore zeolite catalysts for furfural synthesis from xylose and switchgrass in a γ-valerolactone/water solvent. J. Mol. Catal. A Chem..

[44] Gallo J.M.R., Alonso D.M., Mellmer M.A., Yeap J.H., Wong H.C., Dumesic J.A. (2013). Production of Furfural from Lignocellulosic Biomass Using Beta Zeolite and Biomass-Derived Solvent. Top. Catal..

[45] Wang Y., Dai Y., Wang T., Li M., Zhu Y., Zhang L. (2022). Efficient conversion of biomass xylose to furfural over modified zeolite in the recyclable water/n-butanol system. Fuel Process. Technol..

[46] Wang L., Guo H., Wang Q., Hou B., Jia L., Cui J., Li D. (2019). The study of active sites for producing furfural and soluble oligomers in fructose conversion over HZSM-5 zeolites. Mol. Catal..

[47] Hoang P.H., Cuong T.D. (2021). Simultaneous Direct Production of 5-Hydroxymethylfurfural (HMF) and Furfural from Corncob Biomass Using Porous HSO_3_-ZSM-5 Zeolite Catalyst. Energy Fuels.

[48] Shringi N., Sidana C., Rani A. (2023). SO_4_^2−^/SnO_2_-fly ash as bifunctional catalyst for microwave-assisted single-step condensation of 2-naphthol and aromatic aldehydes. Arab. J. Sci. Eng..

[49] Zhang L., Tian L., Sun R., Liu C., Kou Q., Zuo H. (2019). Transformation of corncob into furfural by a bifunctional solid acid catalyst. Bioresour. Technol..

[50] Bakili S., Kivevele T., King’ondu C.K. (2024). Optimization of furfural production from xylose over sulfated titanium-niobium mixed oxides catalyst in biphasic system. Clean. Chem. Eng..

[51] Zhong Y., Liu Y., Wang S., Hou S., Fan Y. (2024). Incorporation of tin oxide nanoparticles on sulfonated carbon microspheres as a bifunctional catalyst for efficient conversion of biomass-derived monosaccharides to 5-Hydroxymethylfurfural and furfural. Ind. Crops Prod..

[52] Fúnez-Núñez I., García-Sancho C., Cecilia J.A., Moreno-Tost R., Pérez-Inestrosa E., Serrano-Cantador L., Maireles-Torres P. (2019). Synergistic effect between CaCl_2_ and γ-Al_2_O_3_ for furfural production by dehydration of hemicellulosic carbohydrates. Appl. Catal. A Gen..

[53] García-Sancho C., Agirrezabal-Telleria I., Güemez M.B., Maireles-Torres P. (2014). Dehydration of D-xylose to furfural using different supported niobia catalysts. Appl. Catal. B Environ..

[54] Chareonlimkun A., Champreda V., Shotipruk A., Laosiripojana N. (2010). Catalytic conversion of sugarcane bagasse, rice husk and corncob in the presence of TiO_2_, ZrO_2_ and mixed-oxide TiO_2_–ZrO_2_ under hot compressed water (HCW) condition. Bioresour. Technol..

[55] García-Sancho C., Rubio-Caballero J.M., Mérida-Robles J.M., Moreno-Tost R., Santamaría-González J., Maireles-Torres P. (2014). Mesoporous Nb_2_O_5_ as solid acid catalyst for dehydration of D-xylose into furfural. Catal. Today.

[56] Kumar Mishra R., Bhooshan Kumar V., Victor A., Neel Pulidindi I., Gedanken A. (2019). Selective Production of Furfural from the Dehydration of Xylose using Zn doped CuO Catalyst. Ultrason. Sonochem..

[57] Moreno-Marrodan C., Barbaro P., Caporali S., Bossola F. (2018). Low temperature continuous flow dehydration of xylose over water-tolerant niobia-titania heterogeneous catalysts. ChemSusChem.

[58] Rakngam I., Osakoo N., Wittayakun J., Chanlek N., Pengsawang A., Sosa N., Butburee T., Faungnawakij K., Khemthong P. (2021). Properties of mesoporous Al-SBA-15 from one-pot hydrothermal synthesis with. Microporous Mesoporous Mater..

[59] Xu S., Pan D., Wu Y., Fan J., Wu N., Gao L., Li W., Xiao G. (2019). Catalytic conversion of xylose and xylan into furfural over Cr^3+^/P-SBA-15 catalyst derived from spent adsorbent. Ind. Eng. Chem. Res..

[60] Yu I.K.M., Tsang D.C.W. (2017). Conversion of biomass to hydroxymethylfurfural: A review of catalytic systems and underlying mechanisms. Bioresour Technol..

[61] Jeanmard L., Rongwong W., Chisti Y. (2025). Biomass-derived levulinic acid as a platform chemical for making diverse products. Biomass Bioenergy.

[62] Yang F., Fu J., Mo J., Lu X. (2013). Synergy of Lewis and Brønsted Acids on Catalytic Hydrothermal Decomposition of Hexose to Levulinic Acid. Energy Fuels.

[63] Hurst G., Teklemariam A., Brierley S., Diaz De Rienzo M.A., Tedesco S. (2025). Lignocellulosic biomass conversion to levulinic acid via acid catalysis: Current methods, opportunities and challenges for self-sustaining biorefineries. Int. J. Thermofluids.

[64] Di Menno Di Bucchianico D., Wang Y., Buvat J.-C., Pan Y., Casson Moreno V., Leveneur S. (2022). Production of levulinic acid and alkyl levulinates: A process insight. Green Chem..

[65] Conti Silva J.A., Grilo L.M., Vasconcelos M.H., Lacerda T.M. (2022). 13—Levulinic acid: Perspectives of its biobased production and most promising derivatives. Production of Top 12 Biochemicals Selected by USDOE from Renewable Resources. Status and Innovation.

[66] Kang S., Fu J., Zhang G. (2018). From lignocellulosic biomass to levulinic acid: A review on acid-catalyzed hydrolysis. Renew. Sustain. Energy Rev..

[67] Nakason K., Kuboon S., Phanthuwongpakdee J., Kraithong W., Jiratanachotikul A., Panyapinyopol B., Kanokkantapong V. (2025). Economic viability and life cycle assessment of levulinic acid and hydrochar production via catalytic hydrothermal process of waste lignocellulosic biomass: A comparison of feedstock types. Case Stud. Chem. Environ. Eng..

[68] Li X., Xu R., Yang J., Nie S., Liu D., Liu Y., Si C. (2019). Production of 5-hydroxymethylfurfural and levulinic acid from lignocellulosic biomass and catalytic upgradation. Ind. Crops Prod..

[69] Šivec R., Grilc M., Huš M., Likozar B. (2019). Multiscale modeling of (hemi)cellulose hydrolysis and cascade hydrotreatment of 5-hydroxymethylfurfural, furfural, and levulinic acid. Ind. Eng. Chem. Res..

[70] Ukawa-Sato R., Hirano N., Fushimi C. (2023). Design and techno–economic analysis of levulinic acid production process from biomass by using co-product formic acid as a catalyst with minimal waste generation. Chem. Eng. Res. Des..

[71] Toftgaard Pedersen A., Ringborg R., Grotkjær T., Pedersen S., Woodley J.M. (2015). Synthesis of 5-hydroxymethylfurfural (HMF) by acid catalyzed dehydration of glucose–fructose mixtures. Chem. Eng. J..

[72] Huang H., Denard C.A., Alamillo R., Crisci A.J., Miao Y., Dumesic J.A., Scott S.L., Zhao H. (2014). Tandem catalytic conversion of glucose to 5-hydroxymethylfurfural with an immobilized enzyme and a solid acid. ACS Catal..

[73] Guo W., Zhang Z., Hacking J., Heeres H.J., Yue J. (2021). Selective fructose dehydration to 5-hydroxymethylfurfural from a fructose-glucose mixture over a sulfuric acid catalyst in a biphasic system: Experimental study and kinetic modeling. Chem. Eng. J..

[74] Zhang X., Murria P., Jiang Y., Xiao W. (2016). Maleic Acid and Aluminum Chloride Catalyzed Conversion of Glucose to 5-(Hydroxymethyl) furfural and Levulinic Acid in Aqueous Media. Green Chem..

[75] Yu I.K.M., Tsang D.C.W., Chen S.S., Ok Y.S., Poon C.S. (2016). Valorization of food waste into hydroxymethylfurfural: Dual role of metal ions in successive conversion steps. Bioresour. Technol..

[76] Sun A., Ying Y., Wang M., Zhu L., Wang Y., Zhang Q., Li L., Cao C., Xu H., Cheng D. (2025). Efficient conversion of fructose to 5-hydroxymethylfurfural by hydrophobic modified SAPO-34 molecular sieve. J. Catal..

[77] Jeong H., Park S.-Y., Ryu G.-H., Choi J.-H., Kim J.-H., Choi W.-S., Lee S.M., Choi J.W., Choi I.-G. (2018). Catalytic conversion of hemicellulosic sugars derived from biomass to levulinic acid. Catal. Commun..

[78] Hu Y., Li H., Wu D., Li L., Hu C., Zhu L. (2024). Boosting catalytic performance of Amberlyst-15 by modulating surface properties for synthesis of 5-hydroxymethylfurfural from high-concentration fructose. Catal. Today.

[79] Wrigstedt P., Keskivali J., Repo T. (2016). Microwave-enhanced aqueous biphasic dehydration of carbohydrates to 5 hydroxymethylfurfural. RSC Adv..

[80] Hou Q., Zhen M., Liu L., Chen Y., Huang F., Zhang S., Li W., Ju M. (2018). Tin phosphate as a heterogeneous catalyst for efficient dehydration of glucose into 5-hydroxymethylfurfural in ionic liquid. Appl. Catal. B Environ..

[81] Weingarten R., Kim Y.T., Tompsett G.A., Fernández A., Han K.S., Hagaman E.W., Conner W.C., Dumesic J.A., Huber G.W. (2013). Conversion of glucose into levulinic acid with solid metal (IV) phosphate catalysts. J. Catal..

[82] Cheng X., Feng Q., Ma D., Chen H., Zeng X., Xing F., Teng J. (2021). Efficient catalytic production of levulinic acid over hydrothermally stable propyl sulfonic acid functionalized SBA-15 in γ-valerolactone-water system. J. Environ. Chem. Eng..

[83] Lee B.W., Seo J.Y., Jeong K., Choi J., Cho K.Y., Cho S., Baek K.-Y. (2022). Efficient production of levulinic acid using metal–organic framework catalyst: Role of Brønsted acid and flexibility. Chem. Eng. J..

[84] Souzanchi S., Nazari L., Rao K.T.V., Yuan Z., Tan Z., Xu C. (2021). 5-HMF production from industrial grade sugar syrups derived from corn and wood using niobium phosphate catalyst in a biphasic continuous-flow tubular reactor. Catal. Today.

[85] Liu B., Ba C., Jin M., Zhang Z. (2015). Effective conversion of carbohydrates into biofuel precursor 5-hydroxymethylfurfural (HMF) over Cr-incorporated mesoporous zirconium phosphate. Ind. Crops Prod..

[86] Van der Graaff W.N.P., Olvera K.G., Pidko E.A., Hensen E.J.M. (2014). Stability and catalytic properties of porous acidic (organo)silica materials for conversion of carbohydrates. J. Mol. Catal. A Chem..

[87] Agirrezabal-Telleria I., Requies J., Gueemez M.B., Arias P.L. (2014). Dehydration of D-xylose to furfural using selective and hydrothermally stable arenesulfonic SBA-15 catalysts. Appl. Catal. B.

[88] Wei W., Wu S. (2018). Experimental and kinetic study of glucose conversion to levulinic acid in aqueous medium over Cr/HZSM-5 catalyst. Fuel.

[89] Ramli N.A.S., Amin N.A.S. (2016). Kinetic study of glucose conversion to levulinic acid over Fe/HY zeolite catalyst. Chem. Eng. J..

[90] Qu H., Liu B., Gao G., Ma Y., Zhou Y., Zhou H., Li L., Li Y., Liu S. (2019). Metal-organic framework containing Brønsted acidity and Lewis acidity for efficient conversion glucose to levulinic acid. Fuel Process. Technol..

[91] Sampath G., Kannan S. (2013). Fructose dehydration to 5-hydroxymethylfurfural: Remarkable solvent influence on recyclability of Amberlyst-15 catalyst and regeneration studies. Catal. Commun..

[92] Dou Y., Zhou S., Oldani C., Fang W., Cao Q. (2018). 5-Hydroxymethylfurfural production from dehydration of fructose catalyzed by Aquivion@silica solid acid. Fuel.

[93] Perveen F., Farooq M., Ramli A., Naeem A., Khan I.W., Saeed T., Khan J. (2023). Levulinic Acid Production from Waste Corncob Biomass Using an Environmentally Benign WO_3_-Grafted ZnCo_2_O_4_@CeO_2_ Bifunctional Heterogeneous Catalyst. ACS Omega.

[94] Michel C., Gallezot P. (2015). Why Is Ruthenium an Efficient Catalyst for the Aqueous-Phase Hydrogenation of Biosourced Carbonyl Compounds?. ACS Catal..

[95] Patel A., Shah A.R. (2021). Integrated lignocellulosic biorefinery: Gateway for production of second generation ethanol and value added products. J. Bioresour. Bioprod..

[96] Roldugina E.A., Kardashev S.V., Maximov A.L. (2024). Hydrogenation of Furfural on Pt- and Pd-Containing Catalysts in an Aqueous Medium. Russ. J. Appl. Chem..

[97] Kumaravel S., Durai M., Kaliyamoorthy S., Kumaravel S., Chandramoorthy C., Avula B., Hasan I., Kim M.-J., Balu K., Ahn Y.-H. (2023). Ru Nanoparticles Supported on Mesoporous Al-SBA-15Catalysts for Highly Selective Hydrogenation of Furfural to Furfuryl Alcohol. ChemistrySelect.

[98] Yuan Q., Zhang D., van Haandel L., Ye F., Xue T., Hensen E.J.M., Guan Y. (2015). Selective liquid phase hydrogenation of furfural to furfuryl alcohol by Ru/Zr-MOFs. J. Mol. Catal. A Chem..

[99] Bardestani R., Biriaei R., Kaliaguine S. (2020). Hydrogenation of Furfural to Furfuryl Alcohol over Ru Particles Supported on Mildly Oxidized Biochar. Catalysts.

[100] Tolek W., Nanthasanti N., Pongthawornsakun B., Praserthdam P., Panpranot J. (2021). Effects of TiO_2_ structure and Co addition as a second metal on Ru-based catalysts supported on TiO_2_ for selective hydrogenation of furfural to FA. Sci. Rep..

[101] Tang Q., Sun X., Duan Z., Xiao Z., Liu X. (2025). Efficient hydrogenation of biomass-derived furfural to tetrahydrofurfuryl alcohol over a non-noble Ni/CeO_2_ catalyst under mild conditions. Mol. Catal..

[102] Zhang Z., Liu R., Huang L., Liu P. (2024). Highly efficient selective hydrogenation of furfural to tetrahydrofurfuryl alcohol over MOF-derived Co-Ni bimetallic catalysts: The effects of Co-Ni alloy and adsorption configuration. J. Catal..

[103] Huang R., Cui Q., Yuan Q., Wu H., Guan Y., Wu P. (2018). Total Hydrogenation of Furfural over Pd/Al_2_O_3_ and Ru/ZrO_2_ Mixture under Mild Conditions: Essential Role of Tetrahydrofurfural as an Intermediate and Support Effect. ACS Sustain. Chem. Eng..

[104] Bruna L., Miquel C.-F., Colliere V., Philippot K., Rosa Axet M. (2022). In Situ Ruthenium Catalyst Modification for the Conversion of Furfural to 1,2-Pentanediol. Nanomaterials.

[105] Kasad M.R., Jackson J.E., Saffron C.M. (2024). Electrocatalytic hydrogenation of the formyl group and heteroaromatic ring in furfural on activated carbon cloth-supported ruthenium. RSC Sustain..

[106] Yan K., Wu G., Lafleur T., Jarvis C. (2014). Production, properties and catalytic hydrogenation of furfural to fuel additives and value-added chemicals. Renew. Sustain. Energy Rev..

[107] Li H., Fang Z., Smith R.L., Yang S. (2016). Efficient valorization of biomass to biofuels with bifunctional solid catalytic materials. Prog. Energy Combust. Sci..

[108] Aldosari O.F., Iqbal S., Miedziak P.J., Brett G.L., Jones D.R., Liu X., Edwards J.K., Morgan D.J., Knight D.K., Hutchings G.J. (2016). Pd–Ru/TiO_2_ catalyst—An active and selective catalyst for furfural hydrogenation. Catal. Sci. Technol..

[109] Kumaravel S., Avula B., Alagarasan J.K., Lee M., Ali W., Khan M.E., Ali S.K., Bashiri A.H., Khan A.U., Balu K. (2024). Development of bimetallic Ru/Ni/SBA-16 catalysts for catalytic hydrogenation of bio-based furfural to 2-methylfuran and furfuryl alcohol. Mater. Chem. Phys..

[110] Sun X., Wen B., Wang F., Zhang W., Zhao K., Liu X. (2024). Research advances on the catalytic conversion of biomass-derived furfural into pentanediols. Catal. Commun..

[111] Upare P.P., Kim Y., Oh K.-R., Han S.J., Kim S.K., Hong D.Y., Lee M., Manjunathan P., Hwang D.W., Hwang Y.K. (2021). Bimetallic Ru_3_Sn_7_ Nanoalloy on ZnO Catalyst for Selective Conversion of Biomass-derived Furfural into 1, 2-Pentanediol. ACS Sustain. Chem. Eng..

[112] Rodiansono, Azzahra A.S., Santoso U.T., Mikrianto E., Suarso E., Sembiring K.C., Adilina I.B., Sunnardiantoe G.K., Afandi A. (2025). Highly efficient and selective aqueous phase hydrogenolysis of furfural to 1,5-pentanediol using bimetallic Ru–SnO_x_/γ-Al_2_O_3_ catalysts. Catal. Sci. Technol..

[113] Kamble P.A., Vinod C.P., Rathod V.K., Kantam M.L. (2023). Hydrogenation of levulinic acid to gamma-valerolactone over nickel supported organoclay catalyst. Catal. Today.

[114] Zhou Y., Wang L., Guo P., Yao G. (2020). Recent advances in the production of γ-valerolactone with liquid hydrogen source. IOP Conf. Ser. Earth Environ. Sci..

[115] Córdova-Pérez G.E., Cortez-Elizalde J., Silahua-Pavón A.A., Cervantes-Uribe A., Arévalo-Pérez J.C., Cordero-Garcia A., Espinosa de los Monteros A.E., Espinosa-González C.G., Godavarthi S., Ortiz-Chi F. (2022). γ-Valerolactone Production from Levulinic Acid Hydrogenation Using Ni Supported Nanoparticles: Influence of Tungsten Loading and pH of Synthesis. Nanomaterials.

[116] Piskun A., Winkelman J.G.M., Tang Z., Heeres H.J. (2016). Support Screening Studies on the Hydrogenation of Levulinic Acid to γ-Valerolactone in Water Using Ru Catalysts. Catalysts.

[117] Wang R., Chen L., Zhang X., Zhang Q., Li Y., Wang C., Ma L. (2018). Conversion of levulinic acid to γ-valerolactone over Ru/Al_2_O_3_–TiO_2_ catalyst under mild conditions. RSC Adv..

[118] Sorokina S.A., Mikhailov S.P., Kuchkina N.V., Bykov A.V., Vasiliev A.L., Ezernitskaya M.G., Golovin A.L., Nikoshvili L.Z., Sulman M.G., Shifrina Z.B. (2022). Ru@hyperbranched Polymer for Hydrogenation of Levulinic Acid to Gamma-Valerolactone: The Role of the Catalyst Support. Int. J. Mol. Sci..

[119] Jaya V.S., Sudhakar M., Kumara S.N., Venugopala A. (2015). Selective hydrogenation of levulinic acid to γ-valerolactone over a Ru/Mg–LaO catalyst. RSC Adv..

[120] Rodríguez V.I., Mendow G., Sánchez B.S., García J.R., Pujro R.A., de Miguel S.R., Veizag N.S. (2023). Ruthenium Catalysts Supported on Hydrothermally Treated Carbon from Rice Husk: The Effect of Reduction Temperature on the Hydrogenation Reaction of Levulinic Acid to γ-Valerolactone. Processes.

[121] Petcuta O.A., Guzo N.C., Bordeiasu M., Nicolaev A., Parvulescu V.I., Coman S.M. (2025). Ru/Beta Zeolite Catalysts for Levulinic Acid Hydrogenation: The Importance of Catalyst Synthesis Methodology. Catalysts.

[122] Rodiansono, Astuti M.D., Mustikasari K., Husain S., Ansyah F.R., Harae T., Shimazu S. (2022). Unravelling the one-pot conversion of biomassderived furfural and levulinic acid to 1,4-pentanediol catalysed by supported RANEY^®®^ Ni–Sn alloy catalysts. RSC Adv..

[123] Lv J., Rong Z., Sun L., Liu C., Lu A.-H., Wanga Y., Qu J. (2018). Catalytic conversion of biomass-derived levulinic acid into alcohols over nanoporous Ru catalyst. Catal. Sci. Technol..

[124] Cui J., Tan J., Zhu Y., Cheng F. (2018). Aqueous Hydrogenation of Levulinic Acid to 1,4-Pentanediol over Mo-Modified Ru/Activated Carbon Catalyst. ChemSusChem.

[125] Lee D., Kim H.U., Kim J.R., Park Y.-K., Ha J.-M., Jae J. (2024). Insights into the structure–activity relationship in aqueous-phase hydrogenation of levulinic acid to 1,4-pentanediol over bimetallic Ru-Re/C catalysts. J. Ind. Eng. Chem..

[126] Cañadas R., Díaz I., Rodríguez M., González E.J., González-Miquel M. (2022). An integrated approach for sustainable valorization of winery wastewater using bio-based solvents for recovery of natural antioxidants. J. Clean. Prod..

[127] Gundekari S., Karmee S.K. (2024). Catalytic Conversion of Levulinic Acid into 2-Methyltetrahydrofuran: A Review. Molecules.

[128] Phanopoulos A., White A.J.P., Long N.J., Miller P.W. (2015). Catalytic Transformation of Levulinic Acid to 2-Methyltetrahydrofuran Using Ruthenium–N-Triphos Complexes. ACS Catal..

[129] Licursi D., Antonetti C., Fulignati S., Giannoni M., Galletti A.M.R. (2018). Cascade Strategy for the Tunable Catalytic Valorization of Levulinic Acid and γ-Valerolactone to 2-Methyltetrahydrofuran and Alcohols. Catalysts.

[130] Upare P.P., Lee M., Lee S.-K., Yoon J.W., Bae J., Hwang D.W., Lee U.-H., Chang J.-S., Hwang Y.K. (2016). Ru nanoparticles supported graphene oxide catalyst for hydrogenation of bio-based levulinic acid to cyclic ethers. Catal. Today.

[131] Menegazzo F., Ghedini E., Signoretto M. (2018). 5-Hydroxymethylfurfural (HMF) Production from Real Biomasses. Molecules.

[132] Post C., Maniar D., Voet V.S.D., Folkersma R., Loos K. (2023). Biobased 2,5-Bis(hydroxymethyl)furan as a Versatile Building Block for Sustainable Polymeric Materials. ACS Omega.

[133] Li M., Zheng T., Lu D., Dai S., Chen X., Pan X., Dong D., Weng R., Xu G., Wang F. (2023). Facet effect on the reconstructed Cu-catalyzed electrochemical hydrogenation of 5-hydroxymethylfurfural (HMF) towards 2,5-bis(hydroxymethy)furan (BHMF). J. Energy Chem..

[134] Huang R., Yuan S., Chen B., Yang Z., Tian Y., Li Z., Lin L., Zeng X. (2024). Selective conversion of 5-hydroxymethylfurfural to 2,5-bis(hydroxymethyl)tetrahydrofuran over Ni-NC/SiO_2_ at room temperature. Chem. Eng. Sci..

[135] Jain A.B., Vaidya P.D. (2016). Kinetics of Catalytic Hydrogenation of 5-Hydroxymethylfurfural to 2,5-bis-Hydroxymethylfuran in Aqueous Solution over Ru/C. Int. J. Chem. Kinet..

[136] Rodríguez-Padrón D., Perosa A., Longo L., Luque R., Selva M. (2022). Tuning the Selectivity of the Hydrogenation/Hydrogenolysis of 5-Hydroxymethylfurfural under Batch Multiphase and Continuous-Flow Conditions. ChemSusChem.

[137] Fulignati S., Antonetti C., Licursi D., Pieraccioni M., Wilbers E., Heeres H.J., Galletti A.M.R. (2019). Insight into the hydrogenation of pure and crude HMF to furan diols using Ru/C as catalyst. Appl. Catal. A Gen..

[138] Wang T., Zhang J., Xie W., Tang Y., Guo D., Ni Y. (2017). Catalytic Transfer Hydrogenation of Biobased HMF to 2,5-Bis-(Hydroxymethyl)Furan over Ru/Co_3_O_4_. Catalysts.

[139] Kashyap P., Jędrzejczyk M., Akhgar M., Aubrecht J., Kubička D., Keller N., Ruppert A. (2025). Ru/TiO_2_ Catalyzed High-Yield Synthesis of Furanic Diols by 5-Hydroxymethylfurfural Hydrogenation with Switchable Selectivity. ChemSusChem.

[140] Mishra D.K., Lee H.J., Truong C.C., Kim J., Suh Y.-W., Baek J., Kim Y.J. (2020). Ru/MnCo_2_O_4_ as a catalyst for tunable synthesis of 2,5-bis(hydroxymethyl)furan or 2,5-bis(hydroxymethyl)tetrahydrofuran from hydrogenation of 5-hydroxymethylfurfural. Mol. Catal..

[141] Sindhu R., Binod P., Pandey A., Ankaram S., Duan Y., Awasthi M.K. (2019). Chapter 5-Biofuel Production From Biomass: Toward Sustainable Development. Current Developments in Biotechnology and Bioengineering.

[142] Dutta S., Mascal M. (2014). Novel Pathways to 2,5-Dimethylfuran via Biomass-Derived 5-(Chloromethyl)furfural. ChemSusChem.

[143] Yan L., Zhang Q., Deng W., Zhang Q., Wang Y., Song C. (2020). Catalytic valorization of biomass and bioplatforms to chemicals through deoxygenation. Advances in Catalysis.

[144] Hu L., Tang X., Xu J., Wu Z., Lin L., Liu S. (2014). Selective Transformation of 5-Hydroxymethylfurfural into the Liquid Fuel 2,5-Dimethylfuran over Carbon-Supported Ruthenium. Ind. Eng. Chem. Res..

[145] Dong Z., Zhanga Y., Xia H. (2024). Selective hydrogenolysis of 5-hydroxymethylfurfural to 2,5-dimethylfuran with high yield over bimetallic Ru–Co/AC catalysts. RSC Adv..

[146] Zu Y., Yang P., Wang J., Liu X., Ren J., Lu G., Wang Y. (2014). Efficient production of the liquid fuel 2,5-dimethylfuran from 5-hydroxymethylfurfural over Ru/Co_3_O_4_ catalyst. Appl. Catal. B Environ..

[147] Buta J.G., Dame B., Ayala T. (2024). Nitrogen-doped ordered mesoporous carbon supported ruthenium metallic nanoparticles: Opportunity for efficient hydrogenolysis of biomass-derived 5-hydroxymethylfurfural to 2,5-dimethylfuran by catalytic transfer hydrogenation. Heliyon.

[148] Insyani R., Barus A.F., Gunawan R., Park J., Jaya G.T., Cahyadi H.S., Sibi M.G., Kwak S.K., Verma D., Kim J. (2021). RuO_2_–Ru/Hβ zeolite catalyst for high-yield direct conversion of xylose to tetrahydrofurfuryl alcohol. Appl. Catal. B Environ..

[149] Wang N., Chen Z., Liu L. (2018). Acid catalysis dominated suppression of xylose hydrogenation with increasing yield of 1,2-pentanediol in the acid-metal dual catalyst system. Appl. Catal. A Gen..

[150] Ren H.-F., Zhu D., Li J.-F., Liu C.-L., Yang R.-Z., Dong W.-S. (2019). One-pot conversion of carbohydrates into γ-valerolactone under the coordination of heteropoly acid based ionic liquid and Ru/ZrO_2_ in water media. Chem. Technol. Biotechnol..

[151] Duan Y., Zhang J., Li D., Deng D., Maa L.-F., Yang Y. (2017). Direct conversion of carbohydrates to diol by the combination of niobic acid and a hydrophobic ruthenium catalyst. RSC Adv..

